# Understanding the Mechanism of Cardiotoxicity Induced by Nanomaterials: A Comprehensive Review

**DOI:** 10.1002/smsc.202400498

**Published:** 2025-02-20

**Authors:** Zaiyong Zheng, Shuang Zhu, Xiaobo Wang, Haoran Wu, Min Fu, Houxiang Hu, Zhanjun Gu, Chunxiang Zhang

**Affiliations:** ^1^ Department of Cardiology Key Laboratory of Medical Electrophysiology Ministry of Education, Basic Medicine Research Innovation Center for Cardiometabolic Diseases Ministry of Education, The Affiliated Hospital of Southwest Medical University Southwest Medical University Luzhou 646000 China; ^2^ Academician Workstation and Department of Cardiology Affiliated Hospital of North Sichuan Medical College Nanchong 637000 China; ^3^ CAS Key Laboratory for Biomedical Effects of Nanomaterials and Nanosafety Institute of High Energy Physics Beijing 100049 China; ^4^ Spallation Neutron Source Science Center Institute of High Energy Physics Dongguan 523803 China; ^5^ College of Materials Science and Optoelectronic Technology University of Chinese Academy of Sciences Beijing 100049 China

**Keywords:** bibliometric analyses, cardiovascular health, nanomaterials, nanotoxicities

## Abstract

Nanomaterials have been vastly used in daily life. However, owing to their unique properties, nanomaterials also show potential side effects. Among the various organs affected by nanomaterials, the circulatory system stands out as particularly vulnerable, drawing additional attention to its cardiac toxicity. To address the cardiovascular nanotoxicity and further promote the safe use of nanotechnology, a comprehensive review of the cardiotoxicity induced by nanomaterials is provided. The review begins by identifying the current research trends and hotspots in this field via a bibliometric analysis. Subsequently, based on the objectively obtained research hotspots, the mechanism of cardiovascular nanotoxicity, including exposure route, membrane injury, ion disturbance, oxidative stress, inflammation, and cell death, is reviewed and discussed. Finally, current strategies for the mitigation of nanotoxicity are also proposed. The objective of this review is to assist readers in understanding the mechanism of cardiotoxicity induced by nanomaterials and to facilitate their safe application for human health.

## Graphical abstract

This review provides a bibliometric analysis for understanding the study of nanotoxicity on cardiovascular health. It begins by outlining the pathways through which nanomaterials enter the cardiovascular system, followed by a summary of the mechanisms underlying nanomaterial‐induced cardiotoxicity. Finally, strategies for mitigating cardiovascular toxicity and future research directions are explored.

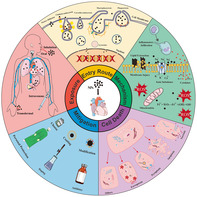

## 1 Introduction

With the rapid advances in nanotechnology, nanomaterials have been employed in various fields including industry, agriculture, and medicine. The unique properties of nanomaterials offer enormous potential, yet they introduce challenges to health and safety. Nanotoxicity can be derived from environmental pollutants and pharmaceutical materials. For instance, with the ability to precisely manipulate and control matter at the nanoscale, nanotechnology can provide diagnostic and therapeutic tools that operate on the same scale as biological molecules.^[^ ^1^ ^]^ As we previously reported, nanomaterials have been widely used in drug delivery, tissue engineering, nanosensors, and nanoimaging applications related to cardiovascular diseases (CVD).^[^ ^2^ ^]^ Meanwhile, the number of drugs containing nanomaterials submitted to the U.S. Food and Drug Administration (FDA) has been increasing since 1973.^[^ ^3^, ^4^, ^5^ ^]^ However, only a few of them have been approved for market because nanotoxicity, especially cardiovascular toxicity, remains a major barrier.^[^ ^6^, ^7^ ^]^

Many nanomedicines have even failed during clinic trails stages, and it's reported that 21% of cancer nanomedicine failed in phase 2 trails, primarily due to nanotoxicity.^[^ ^8^ ^]^ At the same time, as more nanoproducts are being used, the risk of exposure to nanomaterials in our daily life is increasing. Therefore, it's important to explore the mechanism of nanotoxicity. Epidemiological studies have provided strong evidence that both environmental pollutants and pharmaceutical nanomaterials could increase the risk of CVD.^[^ ^9^, ^10^ ^]^ Once entering systematic circulation, nanoparticles (NPs) inevitably interact with the cardiovascular system and impose toxicity across species.^[^ ^11^ ^]^ Different from other organisms, cardiovascular systems, especially myocardium, have limited repair and regeneration capabilities, making it vulnerable to nanotoxicity. From 1994 to 2006, about 45% withdrawn medications were associated with cardiotoxicity.^[^ ^12^ ^]^ Therefore, exploring the cardiotoxicity of nanomaterials is crucial for their development and clinical application in the future.

Since nanomaterials interact with organisms at the atomic or molecular level, nanotoxicity differs in certain aspects from traditional material toxicology, which focuses on the interaction of substances in molecular or ionic form with organisms.^[^ ^13^ ^]^ With unique properties like the size and surface effects, nanotoxicity does not follow a simple dose‐dependent manner. Furthermore, the complexity of nanomaterials makes it difficult to extrapolate the toxicity of one type of NP from another known type.^[^ ^14^ ^]^ Thus, in this review, we present a comprehensive analysis of nanotoxicity in cardiovascular systems based on the specific properties of nanomaterials. Bibliometric analysis was employed to identify current research focus and trends from a holistic perspective. Furthermore, the subsequent section offers a detailed review of the cardiotoxicity induced by nanomaterials with concrete examples. In conclusion, we present our insights and prospects regarding the implications of nanomaterials on cardiotoxicity. This work aims to provide a comprehensive review of the current evidence of this field. We also seek to draw more attention and attract more researchers to explore the mechanisms of cardiotoxicity induced by nanomaterials, ultimately promoting the development of safe and effective nanomedicines.

## 2 Research Methodology

### 2.1 Methods and Data Analysis

#### 2.1.1 Literature Collection

Documents concerning nanomaterials and cardiotoxicity were acquired from the Web of Science (WoS) core collection, which is the world's oldest, most widely used and authoritative database of research publications and citations.^[^ ^15^ ^]^ It has a selective citations index of scientific and scholarly publishing journals, books, and data compilations, covering all the research related to nanotechnology and toxicity. Our aim was to identify articles that focus on hazards of nanomaterials to cardiovascular health. The recruiting criteria are shown in Figure S1, Supporting Information. Prior to conducting the formal search, the indexing time was set to cover the period from “1900‐01‐01 to 2024‐05‐23”. First, we used search terms of “nano*” and “quantum dot* ” to locate documents related to nanomaterials. Excluded studies did not focus on nanomaterials, additional terms like “nanogram concentration*” and “nanomolar concentration*” were precluded in search query #1, thus yielding 2 738 935 results. Next, we used terms such as “cardio* toxicit*”, “Myocard* toxicit*”, “cardiotoxicity”, and “heart toxicit*” in search query #2 to identify research related to cardiotoxicity. Combining search query #1 and #2 produced 2967 documents. Furthermore, after examining the title and abstract of each document, it was found that studies focusing on using nanomaterials to reduce the cardiotoxicity of anticancer drugs are included in this recruitment, which is not in line with the topic of our work. To address this, we created search query #3, incorporating terms like “doxorubicin*” and “adriamycine*” to exclude such studies. Additionally, to avoid incomplete document types such as meeting abstracts and letters, we restricted the search to “Article” or “Review.” A language filter was also applied to limit the results to studies published in English. After applying these filters, a total of 1856 documents were included, consisting of 1538 articles and 318 reviews.

#### 2.1.2 Data Analysis

The innate analysis platform of WoS and bibliometric software VOSviewer (Leiden University's Centre for Science and Technology Studies, version 1.6.17) were used to analyze the distribution of this field across different countries/regions, journals, institutions, and the co‐occurrence of keywords. Through WoS analysis we can uncover the most productive countries and journals in this field. A keywords co‐occurrence network was constructed using VOSviewer to identify the hot research topics and trends in this field. Combined with these results, we provided a comprehensive review of the mechanisms underlying nanomaterial‐induced cardiotoxicity.

### 2.2 Document Characteristics and Annual Trends

**Figure 1:**
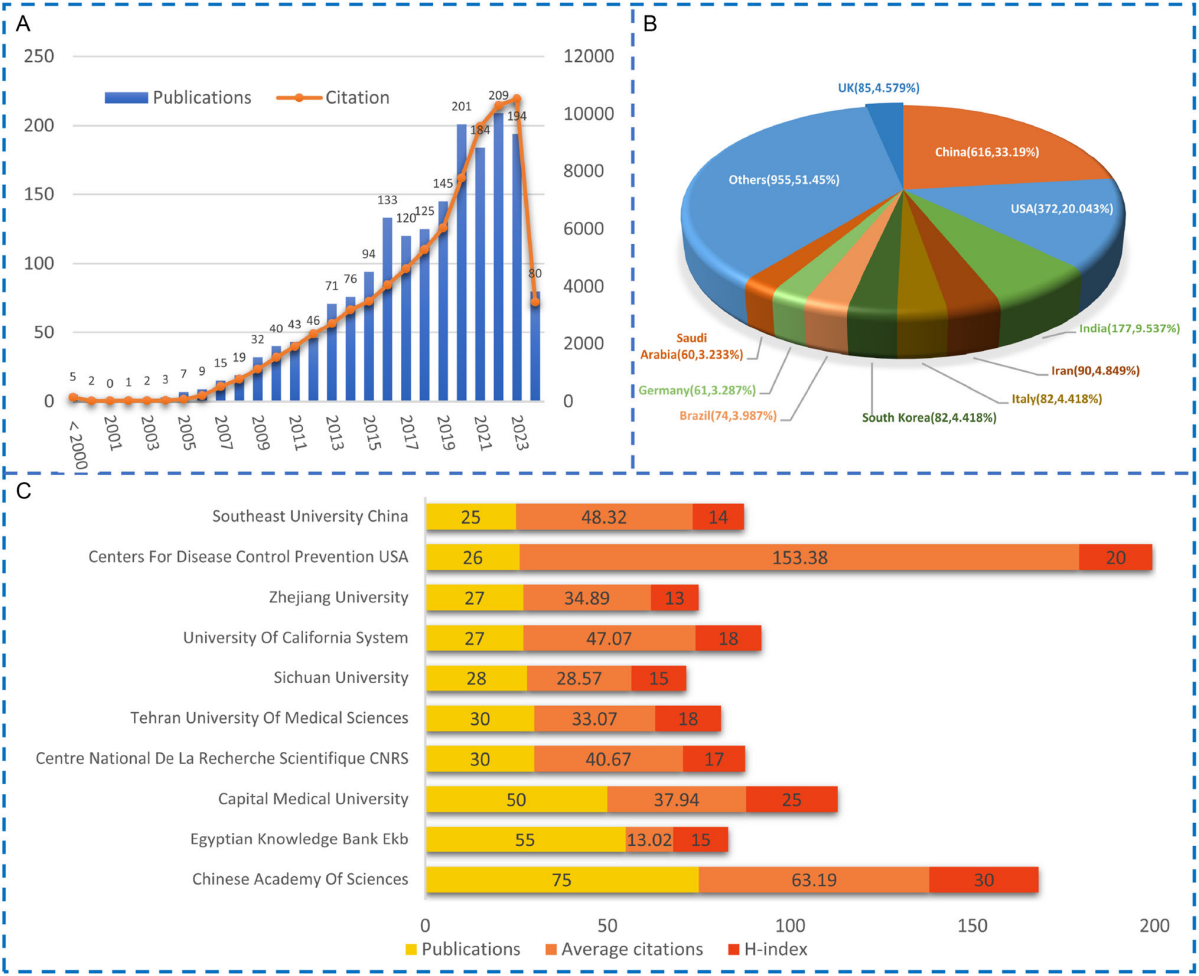
A) Annual growth of publications and citations in literature regarding cardiotoxicity induced by nanomaterials. B) Distribution of publications by countries/regions. The numbers in the brackets represent publication number, percentage, with the percentage indicating the proportion of the publication number on a global scale. C) The most contributing institutes in the field of cardiotoxicity induced by nanomaterials.

A total of 1,898 documents were retrieved as of May 23, 2024. These included 1538 articles, 318 review articles, 3 meeting abstracts, and 39 other types of articles. Note that only original articles and reviews (total 1856 documents) were utilized in our following. The history of this field can be traced back to 1994. The number of documents published in this field has been increasing year by year and has rapidly increased since entering the 21st century. The citations in this field also continue to increase (**Figure** **1**A). These all indicate that researchers continue to pay increasing attention to nanotoxicity in the heart among the research community.

### 2.3 Leading Countries, Regions, and Institutions

Countries all over the world are active in this field. Herein, we present the percentage (in terms of amount) of published literature from different countries all over the world (Figure 1B). We have provided the H‐index of each institution. The H‐index, developed by J. E. Hirsh reflects the productivity based on publications and citations.^[^ ^16^ ^]^ The value of the H‐index is equal to the number of papers (N) in the list that have N or more citations.^[^ ^17^ ^]^ Note that citations and H‐index are major indicators reflecting the influence of a specific country or institute in this research field. It is obvious that North American, Asian‐Pacific, and European countries contribute large numbers of documents in this field. China is the most productive country, with 616 documents, 21 975 citations, and a 72H‐index. The USA provided 372 documents in this field with 28 951 citations and an 80H‐index, indicating that USA has the greatest influence in this field. The following countries are India (177 publications), Iran (90 publications), the UK (85 publications), Italy (82 publications), South Korea (82 publications), Brazil (74 publications), Germany (60 publications), and Saudi Arabia (58 publications). In regard to institution ranking (Figure 1C), the Chinese Academy of Sciences occupies the top institution with 75 documents (4739 citations, 30H‐index), followed by the Egyptian Knowledge Bank (EKB) with 55 documents (716 Citations, 15H‐index).

### 2.4 Key Research Topics in Nanotoxicity and Cardiovascular Health

**Figure 2:**
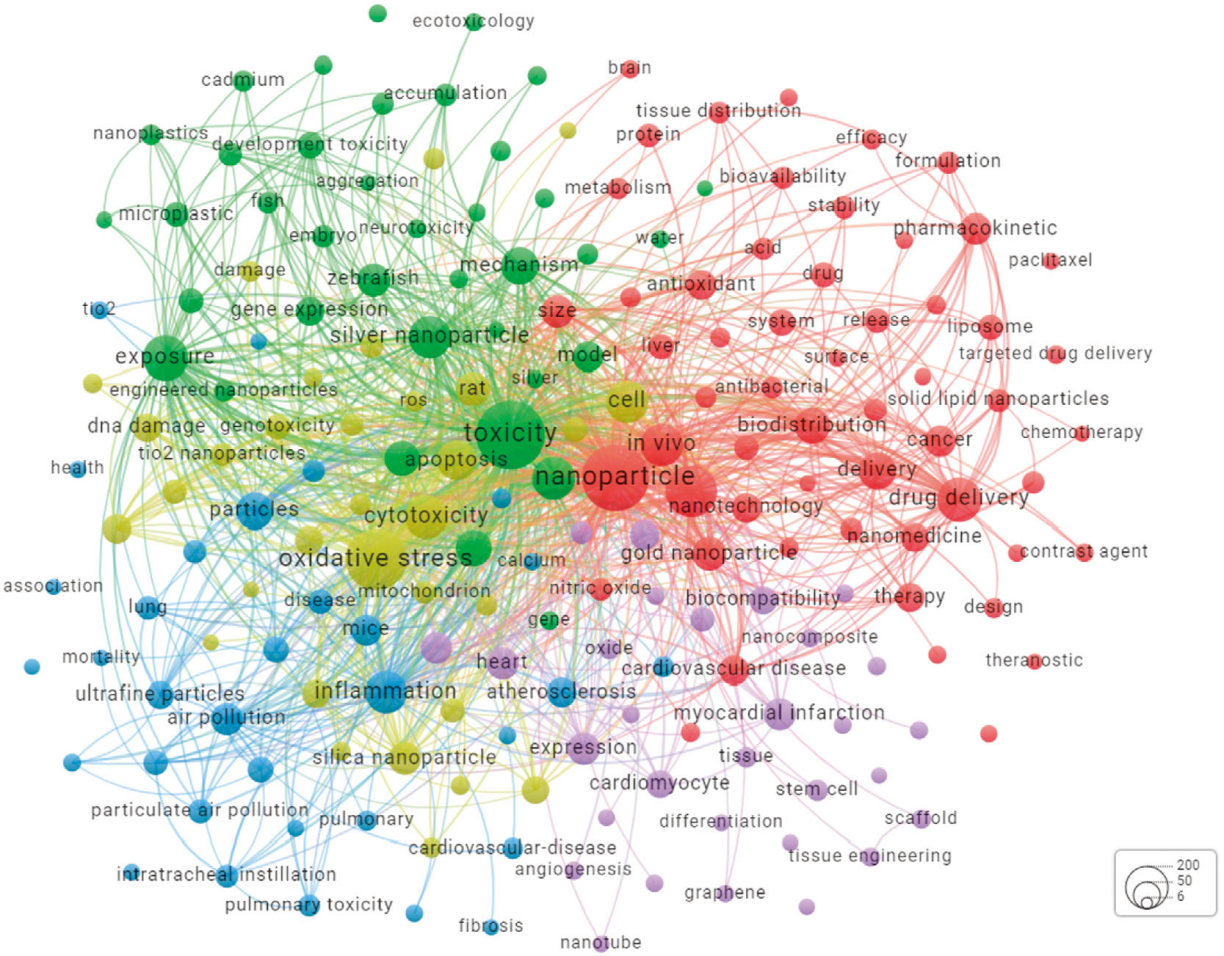
Co‐occurrence analysis of all node sizes indicates the occurrence frequency of the word (online version: https://tinyurl.com/22z7qxmd).

Keywords co‐occurrence maps provide insights into research topics in this field. **Figure** **2** illustrates the utilization of author keywords and Keywords Plus to construct a keywords co‐occurrence map. Keywords Plus is generated using a special algorithm unique to the Clarivate database.^[^ ^18^ ^]^ Derived from the titles of references cited in our research, Keywords Plus serves as an expansion of author keywords, enhancing the ability to reflect the core content of a work.^[^ ^19^ ^]^ After a thorough cleanup (refer to Table S1, Supporting Information), a total of 189 keywords with occurrences exceeding 15 times were identified from 1856 publications.

As depicted in Figure 2, the top ten most frequently occurring keywords were toxicity (565 times), nanoparticle (529 times), oxidative stress (417times), in vitro (275 times), exposure (195 times), drug delivery (191 times), cardiac toxicity (177 times), and cell (170 times). In addition, all keywords were categorized into five clusters, red, green, blue, yellow, and purple.

The red cluster (56 terms) focuses on nanomaterial pharmacokinetics and biodistribution, as indicated by major keywords including drug delivery/release, therapy, in vivo, in vitro, and biodistribution. NPs types associated with this cluster are gold NPs, iron oxide NPs, liposome, and magnetic NPs. The green cluster (38 terms) focuses on exposure toxicity of nanomaterials. Keywords including toxicity, exposure, development toxicity, accumulation, embryotoxicity, neurotoxicity, and ecotoxicology are identified. In addition, nanomaterial types including silver NPs, microplastics, graphene oxides, nanoplastics, and polystyrene NPs and animal models including zebrafish (*Danio rerio*), zebrafish embryo, and fish are associated with exposure toxicity research. The blue cluster (33 terms) focuses on nanoair pollutant‐related inflammation nanotoxicity. Keywords including air pollution, inflammation, particles, toxicology, intratracheal instillation, pulmonary toxicity, and walled carbon nanotubes are observed. Air pollutants including ultrafine particles (UFPs), diesel exhaust particles, and particulate air pollution are widely studied. The yellow cluster (32 terms) focuses on the mechanism of oxidative stress. Keywords including oxidative stress, cytotoxicity, genotoxicity, apoptosis, autophagy, DNA damage, antioxidant, mitochondrial damage, reactive oxygen species (ROS), and NF‐κB are assigned to this cluster. Finally, the purple cluster (30 terms) focuses on the therapeutic purpose of NPs in different types of CVDs such as myocardial infarction, tissue engineering, scaffold, and mesenchymal stem cells.

It becomes apparent that current research on cardiovascular nanotoxicity primarily focuses on the exposure, biodistribution, and toxicity mechanisms, including oxidative stress, inflammation, and various types of cell death. Compared to the extensive research on nanomaterials, studies on cardiotoxicity induced by nanomaterials are still in their early stages. Given that cardiotoxicity is a major obstacle to the clinical use of nanomaterials, it is crucial to explore the underlying mechanisms. Therefore, in the following sections, we first review the pathways through which nanomaterials enter the heart and their subsequent distribution in cardiomyocytes. We then discuss the mechanisms by which nanomaterials induce toxicity in the heart, focusing on oxidative stress, inflammation, and cell death. To address these challenges, we also propose strategies for mitigating cardiotoxicity associated with nanomaterials. Finally, we provide an overview of the limitations and future prospects in this field.

## 3 Routes of Nanomaterial Entry into the Heart

To protect the body's internal environment from external factors, barrier systems including the skin, blood–brain barrier, immune system, and cell membrane have evolved to regulate substances exchange. As an internal organ, the heat is shielded from direct exposure to the external environment. Therefore, NPs must traverse these protective barriers and reach the heart. First, nanomaterials enter the human body via four main routes: oral, inhalation, transdermal, and intravenous administration. After oral administration, nanomaterials will experience gastrointestinal tract, intestinal epithelium, and hepatic first‐pass effects.^[^ ^20^, ^21^ ^]^ In the inhalation route, nanomaterials diffuse through the thick mucus to reach the epithelial cells and then absorb into the blood flow.^[^ ^22^, ^23^ ^]^ In the transdermal route, nanomaterials penetrate the skin and subsequently enter bloodstream.^[^ ^24^, ^25^ ^]^ For intravenous injection, nanomaterials can directly enter the bloodstream. Although there are different routes, nanomaterials will undergo passive diffusion and endocytosis as they enter bloodstream, except intravenous injection. Once they enter blood circulation, the nonspecific interaction between NPs and plasma proteins results in the formation of a protein corona.^[^ ^26^ ^]^ Therefore, the NPs that cells encounter are coated with various protein corona, rather than bare NPs. The physicochemical (such as size, shape, roughness, and porosity)^[^ ^27^, ^28^, ^29^, ^30^ ^]^ and chemical surface parameters (such as hydrophobicity, charge, chirality, and click chemistry)^[^ ^31^ ^]^ of NPs contribute to the different composites of protein coronas. Furthermore, the protein corona varies over different times,^[^ ^32^, ^33^, ^34^ ^]^ entry routes,^[^ ^35^, ^36^ ^]^ and physiological conditions.^[^ ^37^, ^38^, ^39^ ^]^ Subsequently, the protein corona plays a pivotal role in shaping the biological behavior of NPs, such as absorption, distribution, metabolism, excretion, and toxicity.^[^ ^40^, ^41^, ^42^, ^43^ ^]^ Following blood circulation, NPs disseminate throughout the entire body, penetrating across the vasculature and reaching the heart extracellular matrix. NPs cross the vessel endothelium through the transcellular pathway and paracellular pathway. However, as the capillaries within the heart are continuous capillaries with ≈4 nm endothelial gap and low permeability,^[^ ^44^, ^45^ ^]^ the majority of nanomaterials should penetrate endothelial cells rather than leak through the gap between cells (paracellular pathway).^[^ ^46^, ^47^ ^]^ However, it is reported that certain NPs such as Si NPs^[^ ^48^ ^]^ and TiO_2_ NPs^[^ ^49^ ^]^ can disrupt vascular endothelial cadherin (VE‐cadherin) and stretch the gaps between endothelial cells by up to ≈1000 times,^[^ ^49^ ^]^ potentially resulting in more NPs leak through the paracellular pathway. This may also contribute to tissue edema of acute injury after exposure to nanomaterials.

During the process of absorption, entering the bloodstream, and reaching the heart, nanomaterials will repeatedly undergo passive diffusion or endocytic pathways. Each of these processes may influence the subsequently toxicity of nanomaterials. Nevertheless, exploring the mechanisms of how nanomaterials cross cell membranes and subsequently enter the cytoplasm of the cells is important for both nanotherapy and nanotoxicity.

### 3.1 Passive Diffusion

Passive diffusion refers to the process where nanomaterials cross cell membranes driven solely by concentration and electric gradients, requiring no energy. It has been reported that C_60_ can induce leakage in the plasma membrane.^[^ ^50^ ^]^ Molecular dynamics simulations reveal that C_60_ can directly “jump” into the lipid bilayer through the gap (≈1.2 nm) between lipid head groups.^[^ ^51^ ^]^ Passive diffusion can be categorized into two main types: simple diffusion and facilitated diffusion (via channels or carriers). Nonetheless, only a few substances can pass through the membrane via simple diffusion without encountering any obstacles. Similarly, due to the high selectivity of membrane channels and protein carriers, the number of nanomaterials that can cross membranes though facilitated diffusion is also limited. However, nanomaterials that enter cells through passive diffusion may act as a trigger to disrupt the cellular membrane, leading to more NPs “leaking” in cytoplasm.

### 3.2 Endocytosis

Endocytosis is the main mechanism by which nanomaterials cross the plasma membrane and enter cells, involving a complex process that requires extra energy. Continued efforts have been devoted to utilizing endocytosis to transfer NPs into cardiomyocytes for therapy or diagnosis. Strategies for hijacking endocytosis have been used in laboratories, such as employing cationic polymers to deliver DNA or RNA into cells via endocytosis. After entering cells, various approaches, such as the proton sponge effect, are applied to help cargos escape from lysosomes and release.^[^ ^52^ ^]^ Conversely, preventing unnecessary nanomedicine from entering cells is also a key strategy to minimize the toxicity of nanomaterials.

#### 3.2.1 Phagocytosis

Phagocytosis occurs primarily among immune cells such as neutrophils, dendritic cells, macrophages, and some nonspecialized phagocytes like endothelial cells and fibroblasts, which can internalize particles below 10 μm.^[^ ^53^, ^54^ ^]^ The initial step of phagocytosis involves NPs physically binding to cell surface receptors, such as Fc receptors and scavenger receptors. These receptors initiate signaling pathways to remodel and extend the cell membrane, covering the NPs and forming phagosomes. Mature phagosomes then fuse with lysosomes for the degradation of NPs.

#### 3.2.2 Pinocytosis

Different from phagocytosis, pinocytosis internalizes not only molecules but also fluids. Therefore, pinocytosis is described as “cell drinking,” while phagocytosis is described as “cell eating.” Among different cells, pinocytosis can be classified into macropinocytosis, clathrin‐mediated endocytosis, caveolin‐mediated endocytosis, and clathrin‐ and caveolin‐independent endocytosis.^[^ ^55^ ^]^ Various specific pharmacological inhibitors are used to explore the pathways of nanomaterial uptake.^[^ ^56^, ^57^ ^]^ Micropinocytosis is driven by actin and nonselective uptake molecules larger than 0.2 μm.^[^ ^55^, ^58^ ^]^ Inhibiting macropinocytosis with Cyt‐D and 5‐(N‐ethyl‐N‐isopropyl)‐amiloride led to a 50% and 79% decrease in the uptake of hydroxyapatite (HAp) NPs by mouse atrial myocytes.^[^ ^59^ ^]^ This finding indicates that macropinocytosis is the primary pathway for the internalization of HAp NPs. Zhang et al. developed a charge‐reversal amphiphile lipoplex for DNA delivery(277 nm), which enters cells via macropinocytosis.^[^ ^60^ ^]^

Clathrin‐mediated endocytosis is capable of internalizing NPs with a diameter of 100–350 nm. During this process, vesicles are coated with a lattice‐like network formed by a multitude of proteins.^[^ ^61^ ^]^ The cargo NPs are subsequently transported to endosomal or lysosomal compartments for degradation by lysosomal enzymes.^[^ ^55^ ^]^ With pitstop 2 (a special inhibitor of clathrin‐mediated endocytosis), research has revealed that clathrin‐mediated endocytosis partly contributes to the internalization of TiO_2_ NPs in rat cardiomyoblasts.

Caveolin‐mediated endocytosis is characterized by the formation of flask‐shaped invaginations on the plasma membrane called caveolae, with proteins such as caveolin‐1 participating in this process. Caveolin‐mediated endocytosis uptakes molecules with a diameter of 20–100 nm.^[^ ^56^ ^]^ More importantly, the cargo will be transferred to the smooth endoplasmic reticulum (ER) or to the Golgi complex.^[^ ^62^, ^63^ ^]^ Zhang et al. coated calcium phosphate NPs with chondroitin sulfate and alendronate(CP‐ALN‐CS) to transfer DNA into cells. Pharmacological inhibitors revealed that CP‐ALN‐CS NPs enter cells through caveolin‐mediated endocytosis, with subsequent localization in the Golgi apparatus and ER being detected.^[^ ^64^ ^]^

These internalization mechanisms are not entirely independent and often interact or coexist when NPs enter cells.^[^ ^65^ ^]^ For instance, nanohydroxyapatite enters human umbilical vein endothelial cells (HUVECs) both through caveolin‐mediated endocytosis and through clathrin‐mediated endocytosis and to some extent, through macropinocytosis.^[^ ^66^ ^]^

### 3.3 Intracellular Localization of NPs

Since the structure and internal environment of cellular organelles vary, different NPs accumulate in different parts of the cell, such as the cytoplasm, organelles, or nucleus. The diverse location of NPs often results in variable toxicity manifestations. For instance, NPs that accumulate in mitochondria may disrupt the energy supply and induce oxidative stress, while those that accumulate in the nucleus may disrupt gene expression and embryonic development. NPs can cross the plasma membrane by passive fusion or other mechanisms, leading to direct accumulation in the cytoplasm. In contrast, clathrin‐mediated endocytosis transfers NPs to lysosomes, which have an extremely acidic environment, causing the degradation of most NPs. Caveolin‐mediated endocytosis transfers NPs to the Golgi complex. Consequently, many drug delivery strategies focus on designing nanomedicines that are taken up through caveolae‐mediated pathways and macropinocytosis, which are not exposed to acidic environments and digestive threats. Specialized targeting of cellular organelles NPs is also widely explored and reviewed.^[^ ^67^ ^]^

The nuclear membrane controls the exchange of substances between the cytoplasm and the nucleus, with the nuclear pore complex being the main route for substance exchange; it has a diameter of ≈70 nm.^[^ ^68^ ^]^ Due to its complex structure and high selectivity, only NPs with small diameters, hydrophilic properties, and special target coatings can easily enter the nucleus. Macromolecules larger than ≈40 kDa or with diameters exceeding 10 nm have difficulty crossing nuclear pore complexes.^[^ ^69^, ^70^, ^71^ ^]^ Huo et al. used 2 nm gold NPs to deliver nucleic acid medicines into the nucleus, successfully reducing the expression of target gene.^[^ ^72^ ^]^

Another vital cellular organelle is the mitochondria, the energy factories of cells. Many toxicities of NPs are linked to the disruption of normal mitochondrial function. NPs with high lipid solubility, a positive charge, and coatings with mitochondria‐targeting peptides can easily accumulate in mitochondria^[^ ^73^, ^74^, ^75^, ^76^ ^]^ Coincidentally, due to the high energy demand of the heart, mitochondria are the most abundant organelles in cardiomyocytes. Thus, NPs that tend to accumulate in mitochondria pose a significant threat to cardiovascular health. Air pollutants provide a good example: in a postmortem analysis, exogenous iron‐rich NPs were detected in myocardial mitochondria among individuals residing in areas with high‐iron‐rich air pollution.^[^ ^77^ ^]^ Due to the rarity of human heart samples for experimentation, another study analyzed dog heart tissue in the same city, revealing that exogenous NPs accumulate in myocardial mitochondria from samples collected in areas with high air pollution.^[^ ^78^ ^]^ In HUVECs, exposure to Si NPs for 24 h has resulted in their presence within the mitochondria, accompanied by microstructural damage.^[^ ^79^ ^]^ These observations suggest that NPs can penetrate the cell membrane, access mitochondria, and cause injury.^[^ ^80^ ^]^

## 4 Mechanisms of Nanomaterial‐Induced Cardiotoxicity

Due to their unique properties, the number of nanoproducts has increased rapidly in recent years. The European Union Observatory for Nanomaterials reports that there are ≈2200 existing products containing nanomaterials in the EU, EEA, and Swiss markets, and researchers predict this number will keep increasing.^[^ ^81^, ^82^ ^]^ The special properties of nanomaterials endow them with great biotechnological potential. However, their nanoscale also causes different side effects compared to conventional‐scale counterpart materials, which are not yet fully understood. With the fast‐growing nanotechnology industry, nanotoxicity has attracted increasing attention. The Nanodatabase https://nanodb.dk/en, developed by Department of Environmental Engineering at the Technical University of Denmark, has collected more than 5000 products containing nanomaterials, assigning each product a color code with five dots referring to exposure and hazard potential for humans and environments.^[^ ^83^ ^]^ Due to the exposure to blood and limited repair capacity, the cardiovascular system is particularly vulnerable to nanotoxicity. Therefore, it is important to explore the cardiotoxicity of nanomaterials. Factors such as size, shape, and surface properties (surface charge, surface hydrophobicity, and surface atoms/groups) have a significant effect on nanotoxicity. While the toxicity of manufactured nanomaterials has been widely reported,^[^ ^84^ ^]^ there has been limited focus on the cardiovascular system. Therefore, this section aims to provide a comprehensive review of the cardiotoxicity of nanomaterials. It is important to note that not all nanomaterials exhibit cardiac toxicity; in fact, some are utilized to mitigate the side effects of medications.

### 4.1 Membrane Damage

The plasma membrane serves not only as a mechanical support but also, more importantly, as a critical barrier controlling the exchange of substances and signals, essential for maintaining the necessary differences between the inside and outside of the cell. This plasma membrane is a composite structure comprising lipids, proteins, and sugars, with proportions varying depending on the cell type and its state. Phospholipids, due to their amphiphilic properties, form a lipid bilayer where the hydrophilic heads face outward, and the hydrophobic tails are oriented toward the interior of the membrane. Organelles’ membranes share this structure, so mechanisms that damage the plasma membrane may also affect organelle membranes. In addition to targeting cell membranes,^[^ ^85^ ^]^ nanomaterials acting within the cytoplasm also have the potential to injure cell membranes. The mechanism of membrane injury is a pivotal factor in nanotoxicity.

**Figure 3:**
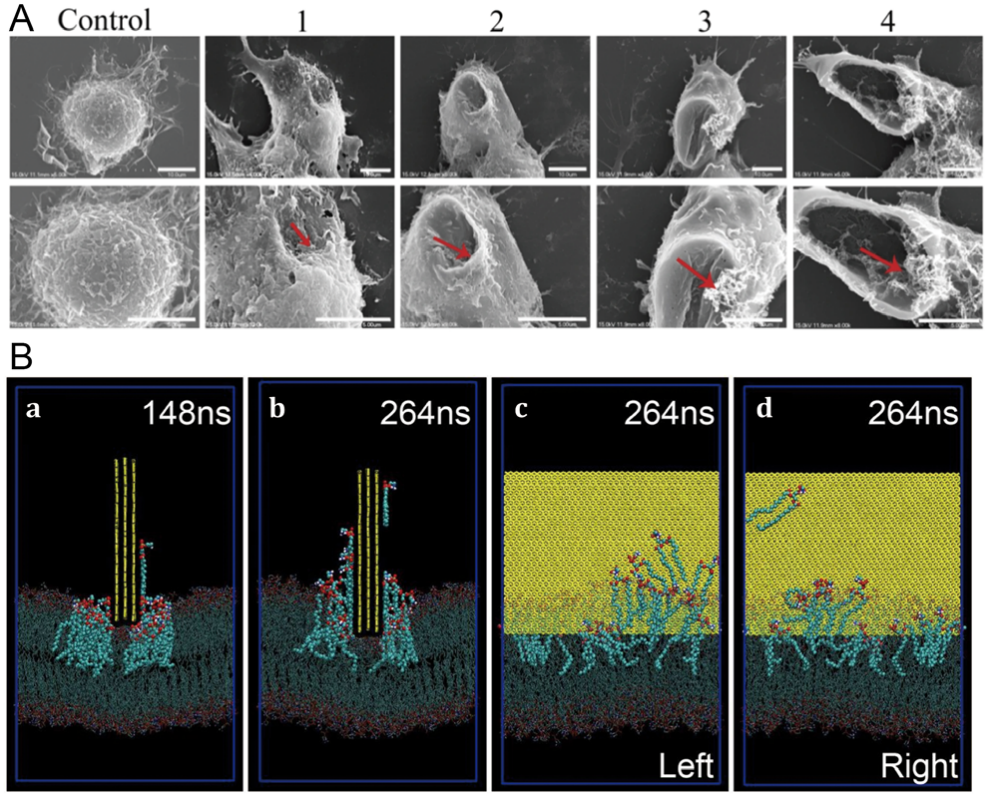
A) Scanning electron microscope images of cell membrane damage (in its final stage, >24 h) incurred by Raw264.7 cells as a result of GO exposure. The subfigure indices 1, 2, 3, and 4 represent progressive degrees of membrane stress observed during different phases of incubation. Scale bar = 5 μm. Reproduced with permission.^[^ ^87^ ^]^ Copyright 2017, Nature Publishing Group. B) All‐atom molecular dynamics reveals a nanomechanical mechanism of lipid extraction caused by enforced mechanical contact for lysosomal permeabilization. a–d) Representative configurations of a three‐layer graphene sheet interacting with a DPPC membrane under a compressive force of 500 pN at 148 ns (a) and 264 ns (b–d). c,d) Left and right views of B, respectively. Reproduced with permission.^[^ ^96^ ^]^ Copyright 2016, PNAS.

After reaching the extracellular matrix, NPs may adhere to the plasma membrane due to various noncovalent interactions, including electrostatic forces and van der Waals forces. Once adhered, NPs may disturb the membrane structure, even causing pores in the membrane. NPs can bind to the membrane within 20s, and subsequent injury can occur within minutes.^[^ ^86^ ^]^ For instance, after “jumping” into the lipid bilayer, C_60_ NPs diffuse within it, inducing local conformational changes and leading to the formation of micropores.^[^ ^51^ ^]^ Another interesting phenomenon is lipid extraction, where NPs with high lipophilicity and strong oxidizing properties irreversibly adsorb to the membrane, extracting lipids due to intermolecular forces. Cell viability significantly decreases after exposure to graphene oxide nanosheets, with electron micrographs revealing pores on the cell membrane. Atomistic simulations show that graphene nanosheets inserted into the phospholipid bilayer extract lipid molecules, reducing lipid density (**Figure** **3**A).^[^ ^87^ ^]^ Lochbaum et al. used supported phosphatidylcholine lipid bilayers to mimic cell membranes and found that gold NPs (2 nm) coated with various cationic ligands promoted irreversible adsorption to the membrane and lipid extraction,^[^ ^88^ ^]^ significantly reducing lipid density and leading to membrane integrity loss and pore formation.^[^ ^89^ ^]^ Once pores form, ions such as Na^+^ and water flood into the cell, causing cell burst and death.^[^ ^90^, ^91^ ^]^

The toxicity of NPs to the plasma membrane is influenced by factors such as chemical composition, size, length, charge, and shape. With decreasing size, the surface area increases, exposing more surface atoms and defects, leading to higher energy and reactive sites.^[^ ^84^ ^]^ Studies show no discernible difference in membrane injury between copper NPs and copper microparticles when surface area is used as the dosing parameter.^[^ ^92^ ^]^ However, NPs with excessively small sizes may directly penetrate the membrane,^[^ ^51^ ^]^ suggesting an optimal NP size range for inducing membrane damage. Atomic force microscopy reveals that SiO_2_ NPs with diameters of 1.2–22 nm cause pores on supported lipid bilayers (≈5 nm), while particle diameters outside this range are covered by the bilayer membrane.^[^ ^93^ ^]^ Carbon nanotubes, recognized early for their toxicity,^[^ ^94^, ^95^ ^]^ can pierce macrophages and accumulate in tissues, inducing repeated inflammation similar to asbestos fibers. Interestingly carbon nanotubes can disrupt membrane structures from within cellular organelles. Upon internalization into lysosomes, long carbon nanotubes interact with internal membranes, extracting lipids and disrupting lysosomes, leading to cell death^[^ ^96^ ^]^ (Figure 3B).

Surface charge also significantly affects nanotoxicity, with positively charged NPs exhibiting more severe toxicity.^[^ ^97^, ^98^ ^]^ Positively charged nanoplastics are more easily internalized into the cytosol, inducing severe injury to neonatal rat ventricular cardiomyocytes.^[^ ^99^ ^]^ This may be due to the electrostatic attraction between the negatively charged plasma membrane and positively charged NPs, facilitating attachment and lipid extraction. NPs can interact with phosphocholine head groups, altering lipid bilayers between fluid and gelled states,^[^ ^100^ ^]^ explaining why TiO_2_ NPs cause membrane leakage even when strongly negatively charged.^[^ ^98^ ^]^

With the rapid development of drug delivery, nanotechnology strategies have been increasingly employed to deliver cargos across the plasma membrane. However, membrane injury is inevitable during the drug delivery process. For example, commercialized nanomaterials like poly‐ethylenimine (such as in vivo‐jetPEI), cationic liposomes (such as Lipofectamine), and other cationic polymers are used to deliver DNA or RNA into cells, often decreasing cell viability and increasing membrane permeability.^[^ ^101^ ^]^ Strategies like microinjection, electroporation, photoporation, and sonoporation also can induce cell membrane damage.^[^ ^102^ ^]^ Yosef et al. fabricated a vertical silicon nanowire array coated with cargos like RNA and proteins. When cells were cultured on this array, the nanowires directly penetrated the membrane to release cargos, causing minimal membrane damage and little effect on cell growth.^[^ ^103^ ^]^ Other strategies include progressive cell membrane mechanoporation using a microfluidic controller to create transient pores with high shear stress for cargo transfer and “hypoosmotic pressure exposure” to reduce osmolality, causing swelling and enhanced membrane permeability for NP penetration.^[^ ^104^ ^]^

NPs intended for cytoplasmic entry have the potential to cause membrane damage. Fortunately, cells possess a repair mechanism as complex as the coagulation system.^[^ ^105^ ^]^ Small injuries (<1 nm) can reseal spontaneously, while large injuries (>100 nm) are repaired via calcium‐dependent exocytosis.^[^ ^106^, ^107^, ^108^ ^]^ If the damage exceeds repair capability, it leads to irreversible injury and cell death.

### 4.2 Ionic Imbalance

**Figure 4:**
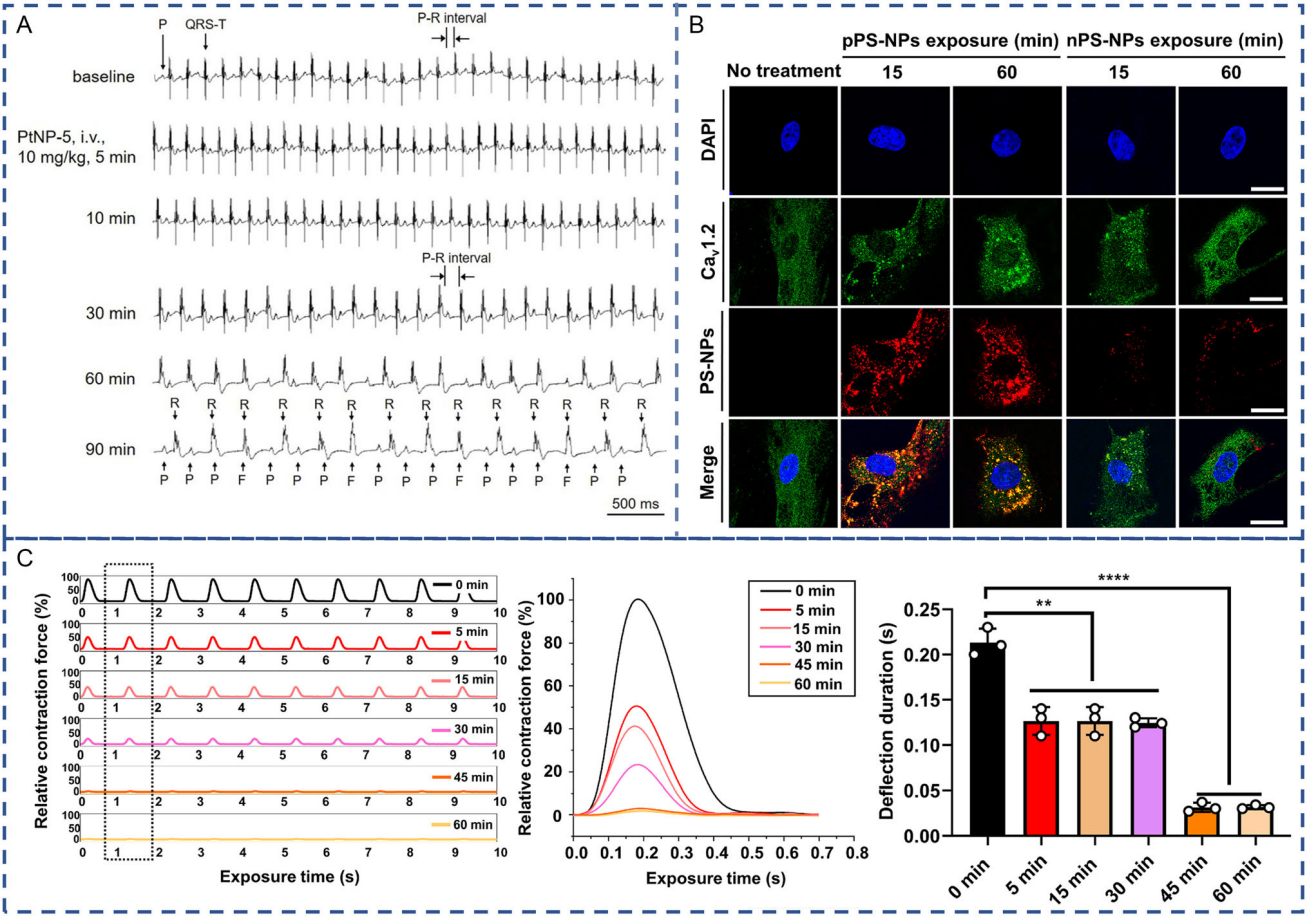
A) Representative electrocardiogram (ECGs) showing the effects of intravenous PtNP‐5 (10 mg kg^−1^) on the heart rate and rhythm of a mouse in vivo. Reproduced with permission.^[^ ^110^ ^]^ Copyright 2021, Dovepress. B) Cells were treated with either nPS‐NPs (red) or pPS‐NPs (red) for two different times under electrical synchronization. Reproduced with permission.^[^ ^99^ ^]^ Copyright 2021, Elsevier. C) Effect of nanoplastic exposure on the collective contractility of cardiomyocytes under electrical stimulation. Reproduced with permission.^[^ ^99^ ^]^ Copyright 2021, Elsevier.

NPs disrupt the balance of ionic homeostasis in myocardial cells through various mechanisms, leading alterations in cardiac rhythm and contractility. The regulated flux of ions, including K^+^, Ca^2+^, Na^+^, and Cl^−^, ensures the generation and spread of myocardial electrical activity and heart rhythms. Disruption of this balance can lead to irregular rhythms. For example, Ag NPs significantly decrease Na^+^ currents (*I*_Na_) and K^+^ currents (*I*_K1_), accelerate the activation of *I*_Na_ channels, ultimately inducing sinus bradycardia, complete A‐V conduction block, cardiac asystole, and death in mice within 20 min of intravenous injection.^[^ ^109^ ^]^ However, research found that Ag^+^ does not induce the same hazardous effects as Ag NPs.^[^ ^109^ ^]^ Similarly, platinum NPs decreased *I*_Na_, *I*_K1_, and *I*_to_ and induced complete atrioventricular conduction block in mice^[^ ^110^ ^]^ (**Figure** **4**A). SiO_2_ NPs significantly increased [Ca^2+^]_i_ in pulmonary artery smooth muscle (SM) cells within just a few seconds of exposure,^[^ ^111^ ^]^ indicating NPs’ ability to disrupt ion balance even without internalization.

Plasma and organelle membranes, as well as ion channels, play crucial roles in maintaining ion balance. NPs can disrupt this balance by damaging membranes and ion channels.^[^ ^112^, ^113^, ^114^ ^]^ Various mechanisms contribute to membrane damage, resulting in ion leakage. NPs can disturb crucial ion channels, such as hERG potassium channel in the heart. ZnO NPs disrupt the hERG current and channel gating, while Zn^+^ ions do not.^[^ ^115^ ^]^ Positive nanoplastics decrease intracellular Ca^2+^ levels and mitochondrial membrane potential, leading to a decrease in contractile force by 50% at 5 min and 97% at 60 min^[^ ^99^ ^]^ (Figure 4C). There was no change in calcium‐ion channel protein levels, but highly overlapping spots of internalized positive nanoplastics and Ca_v_1.2 (Ca^2+^ channel) were observed (Figure 4B). Researchers suggest that positive nanoplastics may interfere with Ca^2+^ by binding to the extracellular portion of the Ca^2+^ channel before internalization and to the cytosolic portion after internalization through strong electrostatic interactions.^[^ ^99^ ^]^ In general, ion channels consist of two components: sensor modules and pore modules. Sensor modules sense various stimuli (such as voltage, chemical signals, and mechanical forces) and regulate the opening and closing of the ion channels, while pore modules provide a physical channel for ion flow. NPs can affect both components of ion channels. Due to their similar size to ion channels, some NPs can block ion channels directly, especially those with a small diameter.^[^ ^116^, ^117^ ^]^ Computer simulations have shown that NPs such as C60 (0.7 nm) and single‐walled carbon nanotubes (SWCNTs, 0.9 nm) can fit into ion channels and obstruct ion flow.^[^ ^118^, ^119^ ^]^ Additionally, residual nickel‐yttrium catalysts used in SWCNT fabrication can release yttrium ions upon exposure which compete with and displace calcium ions from binding sites, inhibiting calcium ion flow. These results highlight that residual impurities can also contribute to nanotoxicity.^[^ ^120^ ^]^

Interfering with the sensor modules is another mechanism by which NPs disrupt ion channels. Sensor modules can be activated by various stimuli, and NPs can affect them in many ways, such as disrupting membrane potential, altering membrane mechanical properties, and interacting with the sensor modules of the ion channel. For example, Gu et al. investigated the effect of MoS_2_ nanoflakes (2.8 nm) on four representative K^+^ channels: KcsA, Kir3.2, K2P2, and the Kv1.2 paddle chimera.^[^ ^121^ ^]^ Electrophysiology results demonstrated that the nanoflake increased K^+^ ion flux in the KcsA channel, blocked K^+^ ion influx in a concentration‐dependent manner in Kir3.2 and Kv1.2 channels, and had no effect on K2P2. Interestingly, molecular dynamics simulations suggested that the mechanism of the MoS_2_ nanoflake's effect on these K^+^ channels is different. The increased K^+^‐ion influx in the KcsA channel was attributed to the interaction of MoS_2_ nanoflakes and the selectivity filter structure, resulting in strong K^+^ leakage. In the Kir3.2 channel, MoS_2_ nanoflakes completely covered the pore modules and effectively blocked K^+^‐ion influx. In the Kv1.2 channel, the MoS_2_ nanoflake binds to the voltage sensor domain of the channel, thereby delaying and disrupting the normal gating process.^[^ ^121^ ^]^ In lipid bilayer membrane models, 5.4 nm Au NPs reduce the activity of gramicidin A (gA) ion channels, and the lifetime of gA channels correlates with alterations in membrane mechanical properties. Results from vibrational spectroscopy and molecular dynamics simulation indicate that these effects are due to the changes in membrane mechanical properties induced by Au NPs, rather than Au NPs entering and blocking the channel.^[^ ^113^ ^]^ Large conductance Ca^2+^‐dependent K^+^ channels (BK_Ca_) are one of the four main K^+^ channels of smooth muscle cells. The contractile function of SM is related to vascular hypercontractility and hypertension. Colloidal Au NPs activate the BK_Ca_ channel, increase the *I*_K_, and finally induce vasorelaxation. This effect can be enhanced by green laser irradiation, potentially due to the interaction of enhanced surface plasmons of Au NPs (by green laser irradiation) and the voltage sensor domain of the BK_Ca_ channel.^[^ ^122^ ^]^ Conversely, polyvinylpyrrolidone‐coated Au NPs increased the current through Ca_v_1.2 and Ca_v_3.1 channels (I_Ba1.2_ and I_Ba3.1_), finally causing vasoconstriction in in vitro rat aorta rings.^[^ ^123^ ^]^

Different NPs may exhibit varying effects on the same ion channel, and these effects differ across different ion channels. NPs may disrupt ion balance through multiple mechanisms simultaneously, affecting both pore and sensor modules of ion channels. For example, C_60_ fullerenes block BK_Ca_ channels, inhibiting net potassium currents and opposing endothelium‐dependent vasorelaxation.^[^ ^124^ ^]^ Copper (Cu) nanomaterials decrease BK_Ca_ channel‐related vasodilation response in aortic rings compared to the Cu‐deficient group but not the Cu carbonate group (representing Cu^2+^).^[^ ^125^ ^]^ In HUVECs, nano‐SiO_2_ increases outward potassium channel activity and channel protein (K_V_1.3), resulting in an increase in the *I*_K_ current and finally promoting proinflammatory cytokine levels and cell death.^[^ ^126^ ^]^ Additionally, NPs can also alter the protein expression of ion channels. For example, in zebrafish embryos, nano‐SiO_2_ decreased the protein expression of calcium channel‐related genes.^[^ ^127^ ^]^ Recent work also reported that exposure to polystyrene nanoplastics can reduce the expression of calcium channels in zebrafish embryos.^[^ ^128^ ^]^

A noteworthy study also reported that NPs can absorb serum ions and lead to cardiotoxicity.^[^ ^129^ ^]^ Similarly, another study reported that polyvinylpyrrolidone‐functionalized silver NPs can impair the function of pacemaker cells, potentially leading to arrhythmia.^[^ ^130^ ^]^ Conversely, benefiting from the unique properties of NPs, numerous strategies have utilized nanomaterials for the remote control of ion channels to treat ion channel‐related diseases.^[^ ^131^ ^]^ Qingbo et al. constructed a chitosan‐desferrioxamine nanosponge to remove excess iron after myocardial infarction, which subsequently reduced oxidative stress and ferroptosis.^[^ ^132^ ^]^

### 4.3 Oxidative Stress

**Figure 5:**
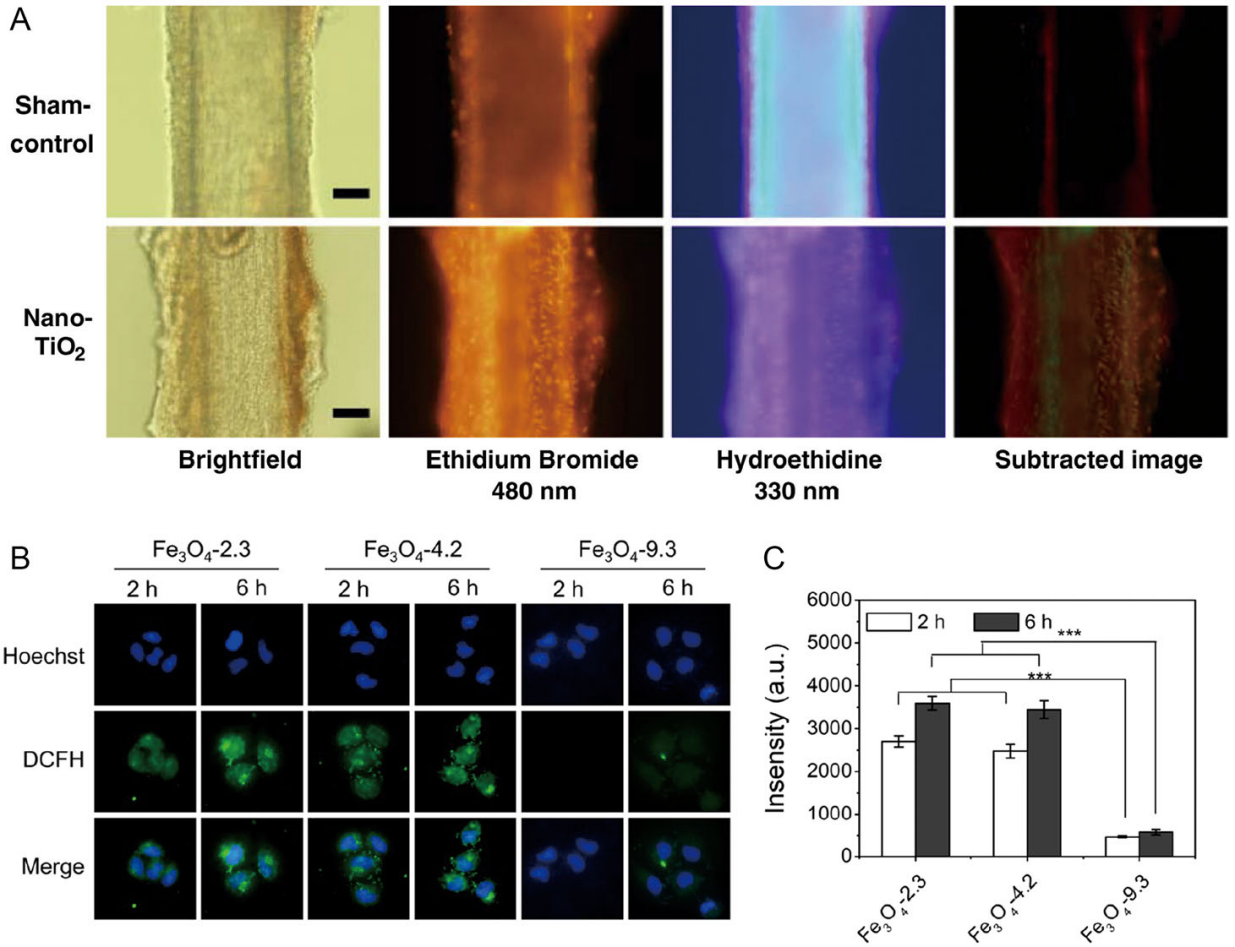
Detection of the generated ROS. A) Representative images of coronary arterioles loaded with DHE from sham‐control and nano‐TiO_2_ exposed rats. Reproduced with permission.^[^ ^139^ ^]^ Copyright 2010, Springer Nature. B) Fluorescence images showing the ROS of MCF‐7 cells after incubation with different‐sized Fe_3_O_4_ with a concentration of 500 μg mL^−1^ for 2 and 6 h. Green fluorescence: DCFH‐DA, an indicator of ROS; blue fluorescence: Hoechst 33 342. Reproduced with permission.^[^ ^146^ ^]^ Copyright 2022, BMC. C) The corresponding fluorescence intensities of DCFH in MCF‐7 cells after incubation with different‐sized Fe_3_O_4_ for 2 and 6 h. Reproduced with permission.^[^ ^146^ ^]^ Copyright 2022, BMC.

Oxidative stress is defined as an excessive ROS in the body, overwhelming the endogenous antioxidant system. ROS are highly reactive oxygen‐containing substances, with primary types including hydrogen peroxide (H_2_O_2_), superoxide (−O_2_), hydroxyl radicals (⋅HO), peroxynitrite (OONO−), nitric oxide (NO), and singlet oxygen (O_2_).^[^ ^133^ ^]^ ROS can be generated in several cellular organelles, such as mitochondria, the ER, and peroxisomes. Due to the heart's continuous motion and high energy expenditure, the mitochondrial respiratory is the major source of intracellular ROS generation in the heart.^[^ ^134^ ^]^ Since ROS can serve as a weapon to eradicate pathogens, immune cells like macrophages and neutrophils also generate substantial amounts of ROS during the inflammatory response. Antioxidant enzymes, including superoxide dismutase (SOD), glutathione peroxidase (GPX), and catalase, work to neutralize the harmful effects of ROS in the human body.^[^ ^135^ ^]^ These antioxidant enzymes are distributed within different organelles.^[^ ^136^ ^]^ The balance between ROS and the antioxidant system maintains the redox state, which is crucial for cellular function. Excessive ROS can interact with various cellular components, destroying their normal structure and leading to DNA/RNA breakage, protein oxidative carbonylation, lipid peroxidation, and membrane destruction.^[^ ^137^ ^]^ Ultimately, these processes contribute to the development of various pathological conditions. Oxidative stress is implicated in various CVD, such as atherosclerosis, myocardial ischemia reperfusion injury, and heart failure. Numerous experiments have also uncovered a correlation between decreased cell viability and ROS generation after exposure to NPs.^[^ ^138^ ^]^ For instance, inhalation of TiO_2_ NPs in rats led to a substantial elevation in ROS levels within coronary arterioles, consequently impairing the vasodilator responses to acetylcholine^[^ ^139^ ^]^ (**Figure** **5**A).

NPs can induce oxidative stress through three primary pathways: generating ROS by themselves, promoting endogenous ROS generation, and inhibiting the antioxidant system. First, due to their high surface reactivity, NPs can directly generate ROS. For example, carbon nanotubes, TiO_2_ NPs, iron‐based NPs, and QDs^[^ ^140^ ^]^ can produce ROS directly and are employed in cancer photodynamic therapy. Fullerene C_60_ can generate ROS in aqueous media under photoirradiation.^[^ ^141^ ^]^ Under light excitation, QDs alone can produce singlet oxygen (^1^O_2_).^[^ ^140^ ^]^ Furthermore, TiO_2_ rutile NPs can generate singlet oxygen under visible light.^[^ ^142^ ^]^ However, another study revealed that even in the dark, TiO_2_ NPs also generate ROS.^[^ ^143^ ^]^ Although it is difficult to directly ascertain ROS generation by NPs in the human body, it is important to consider.

The second mechanism involves disrupting normal biological interactions, thereby promoting excessive endogenous ROS generation. The corresponding ions released from metal NPs and the Fenton reaction are important topics in oxidative stress induced by metal NPs.^[^ ^144^ ^]^ NPs such as CuO NPs and iron‐based NPs can release corresponding ions after administration. After exposure to Fe_2_O_3_ NPs via intraperitoneal injection, researchers found a significant accumulation of ferric ions in mouse hearts.^[^ ^145^ ^]^ The Fenton reaction between Fe^2+^ and H_2_O_2_ converts H_2_O_2_ to a higher oxidative ROS, hydroxyl radical(⋅OH), which cause more severe oxidative damage to cell components. Both SiO_2_ NPs and Fe_2_O_3_ NPs increased in mouse hearts after administration, but ⋅OH was detected only in the Fe_2_O_3_ NPs group.^[^ ^146^ ^]^ Other materials such as copper (Cu), manganese (Mn), molybdenum (Mo), and ruthenium (Ru) can also induce Fenton‐like reactions,^[^ ^144^, ^147^, ^148^ ^]^ leading to cardiac injury. It has been reported that CuO NPs induced oxidative stress and lipid peroxidation (increased MDA levels), causing cell damage and death. The copper ion chelator tetrathiomolybdate alleviates the hazardous effects and decreases MDA induced by CuO NPs, indicating that copper ion release from CuO NPs was involved in CuO NPs‐induced cell injury.^[^ ^149^ ^]^

Third, NPs can not only affect ROS levels but also the antioxidant system. Initially, NPs increase ROS levels and activate the antioxidant system. The antioxidant system eliminates ROS up to a certain level. ROS cause cell injury when the amount of ROS exceeds the antioxidant defense system's capacity. Several studies have revealed that antioxidant systems, such as SOD, increase at the compensated stage but decreased significantly with rising NPs and ROS levels.^[^ ^150^ ^]^ When NPs continue to increase after the compensated stage, oxidative stress turns to decompensated stages, leading to higher ROS levels. SOD is rapidly consumed, resulting in decreased SOD levels.^[^ ^150^, ^151^, ^152^ ^]^ As antioxidant levels decrease, excessive ROS can lead to increased levels of MDA (indicating lipid peroxidation),^[^ ^151^ ^]^ DNA injury, and cell death.^[^ ^153^ ^]^ More importantly, NPs not only consume antioxidant agents but also inhibit the activation of the antioxidant system.^[^ ^154^ ^]^ The mRNA expression levels of Nrf2 decreased, and the downstream antioxidant enzymes Hmox1 and Gpx4 also decreased after exposure to Ag NPs.^[^ ^155^ ^]^

Nanomaterials of different sizes, compositions, doses, and surface chemistries induce various levels of oxidative stress in the heart. In HUVECs, 10 nm Si NPs induced more ROS and decreased more GSH than 25, 50, and 100 nm.^[^ ^156^ ^]^ In rat aortic endothelial cells, SWCNTs increased ROS while decreasing GSH and T‐NOS levels in a concentration‐dependent manner.^[^ ^151^ ^]^ As the concentration of SWCNTs increased, the rise in malondialdehyde (MDA) levels indicated that high dose of SWCNTs induced lipid peroxidation through ROS.^[^ ^151^ ^]^ Compared with SiO_2_ (2.4 nm) and Au (4.1 nm) at the same concentration, Fe_3_O_4_ (2.3 nm) induced more severe oxidative stress both in vivo and in vitro.^[^ ^146^ ^]^ After IV injection, the USPIO (10 μg kg^−1^) group induced significant ROS compared to the saline‐only group, while diesel exhaust particles (400 μg kg^−1^) induced higher ROS and other hazardous effects, such as platelet aggregation and DNA damage.^[^ ^157^ ^]^ The difference in the effect on the redox state between Co NPs and Co^2+^ is higher than between Ni NPs and Ni^2+^.^[^ ^158^ ^]^ Furthermore, NPs with different functional modifications induce different levels of oxidative stress even with the same chemical composition. This phenomenon is also utilized to reduce nanotoxicity. After processing with ozone (O_3_), carbon black gains a negative zeta potential and more functional groups (carbonyls, carboxyl, hydroxyl). This change finally induced severe oxidative stress compared to pure carbon black.^[^ ^159^ ^]^ Reduced graphene oxide (rGO) manufactured from graphene oxide via gamma irradiation contains fewer oxygen‐containing groups and results in different surface properties. After coincubation with H9C2 cells, rGO induced more ROS than GO. This result was also identified in mouse cardiac tissue. With increased doses of absorbed gamma irradiation, rGO induced higher ROS levels than the lower dose group.^[^ ^160^ ^]^ This variation might be due to the functional group change between GO and rGO.^[^ ^156^ ^]^ Another research also found that GO with low oxygen content induces significant dysfunction in isolated rat hypertensive hearts under Langendorff constant‐pressure perfusion condition.^[^ ^161^ ^]^

In contrast, the physical state of the body (especially physiological and pathological scenarios) also affects the toxicity of NPs. Nickel oxide NPs significantly increased mitochondrial O_2_^−^ and nitrite production under pathological conditions (20% cycle stretch and hypoxia) compared to physiological conditions (static and normoxic).^[^ ^162^ ^]^ In spontaneously hypertensive rats, USPIONs introduced more severe oxidative damage to lipids and proteins compared to normotensive rats.^[^ ^163^ ^]^ These results indicate that more attention should be given to the use of USPION products (such as contrast agents, and drug delivery) in hypertensive patients. After co‐exposure to cisplatin (an anticancer drug), cerium oxide NPs induced more oxidative stress, Nrf2 expression, inflammation, and DNA damage.^[^ ^164^ ^]^ With alloxan (provoking oxidative stress), CuO NPs induced significant O_2_‐ levels compared to the CuO NPs‐only group and micro‐CuO group.^[^ ^164^ ^]^ In another study, researchers used alloxan to mimic pathological condition, revealing that low concentrations of TiO_2_ NPs cause oxidative stress and heart function injury but have no effect on oxidative stress markers in healthy rat hearts.^[^ ^165^ ^]^ Therefore, it's important to explore NP toxicity in different pathological scenarios.

The amount of Ag NPs that entered HUVECs was significantly reduced when incubated with N‐acetyl‐l‐cysteine (an oxidation inhibitor). This indicated that ROS induced by NPs, in turn, promote more NP uptake into HUVECs.^[^ ^166^ ^]^ NPs with different properties will locate in different positions and organelles after uptake into cells. This phenomenon can also be utilized for organelle‐targeted drug delivery. Nickel oxide NPs (NiO NPs) increased intracellular ROS and calcium in mitochondria.^[^ ^167^ ^]^ ROS induced by Fe_2_O_3_ NPs (2.3 nm and 4.2 nm) can be detected throughout the cell, including the nucleus, indicating that Fe_2_O_3_ NPs can cross the nuclear membrane and locate in the nucleus^[^ ^146^ ^]^ (Figure 5B,C).

### 4.4 Inflammatory Response

Inflammation is an essential self‐response to harmful stimuli that aims to recognize, neutralize, and eliminate hazardous substances both endogenous and exogenous. It is crucial for protection against harm and for repairing injury. As foreign substances, NPs can induce an inflammatory response via various pathways.^[^ ^168^, ^169^ ^]^ For example, integrated transcriptomics and metabolomics have revealed that nanoplastics induce an inflammatory response in the spleen by disrupting key metabolites and vital genes.^[^ ^170^ ^]^ As previously discussed, nanomaterials can reach the heart via blood flow. Therefore, this section focuses on the inflammation induced by nanomaterials in the heart. The inflammatory response is a complex process characterized by the secretion of inflammatory mediators and the infiltration of inflammatory cells.^[^ ^171^ ^]^

Many inflammatory mediators, such as selectins, integrins, cytokines, chemokines, and acute‐phase proteins, play vital roles in recruiting and guiding circulating leukocytes across the vascular endothelium. These proteins are also widely used to confirm inflammation.^[^ ^172^ ^]^ Selectins, including l‐selectin (leukocytes), *P*‐selectin (endothelial cells, platelets), and E‐selectin (endothelial cells), facilitate the first contact between inflammatory cells and the endothelium of vessel walls, which is a crucial step in recruiting circulating immune cells.^[^ ^173^, ^174^ ^]^ For example, E‐selectin gene expression increased after four hours of exposure to cobalt NPs (Co NPs) in human aortic endothelial cells (HAECs).^[^ ^175^ ^]^ In human EA.hy926 vascular endothelial cells, nanoscale zerovalent iron increased the mRNA expression of E‐selectin.^[^ ^176^ ^]^ Integrin, another group of inflammatory mediators, mediates cell–cell and cell–extracellular matrix adhesion,^[^ ^147^, ^177^ ^]^ adhering to cell adhesion molecules like ICAM‐1, ICAM‐2, and VCAM‐1 on endothelial cells.^[^ ^178^ ^]^ Integrins are essential for the slow movement of leukocytes along the endothelium to find suitable extravasation sites, allowing them to cross the endothelium and reach cardiac tissue.^[^ ^173^ ^]^ Cytokines, secreted by immune cells, act as molecular messengers to recruit leukocytes, modulate their functions, and stimulate the proliferation and differentiation of immune cells.^[^ ^172^ ^]^ Cytokines such as IL‐1, IL‐6, tumor necrosis factor (TNF)‐α, IFN‐γ, and TGF‐β are used to confirm inflammation. After long‐term exposure to TiO_2_ NPs via intragastric administration for 90 days, TNF‐α, IL‐6, and IL‐1β significantly increased in mouse heart tissue.^[^ ^179^ ^]^ Another study showed that UFPs emitted from diesel vehicles at different driving cycles induce different inflammatory responses.^[^ ^180^ ^]^ For example, HUVEC cells exposed to UFPs from an urban dynamometer driving diesel vehicle schedule exhibited higher levels of inflammatory genes like IL‐8, MCP‐1, and VCAM, where UFPs from an idling diesel truck vehicle induced higher oxidative stress but no inflammatory response.^[^ ^180^ ^]^ This difference is attributed to the different chemical compositions of UFPs under various driving schedules. Furthermore, lipophilic organic chemicals adhered to diesel exhaust particles increase IL‐1α, IL‐1β, and MMP‐1 gene expression in endothelial cells.^[^ ^181^ ^]^ Inflammatory chemokines recruit effector leukocytes to sites of infection or damage. IL‐8, MCP‐1, and others are explored in the inflammation induced by NPs in the heart. After exposure to TiO_2_ NPs, primary vascular endothelial cells showed increased mRNA expression of MCP‐1 and VCAM‐1.^[^ ^182^ ^]^ Protein levels of MCP‐1 and IL‐8 were significantly different in primary endothelial cell lines derived from the human aorta (HAECs) and HUVECs after exposure to cobalt NPs and Ti NPs, respectively.^[^ ^175^ ^]^ Interestingly, oxidative stress was not detected in the Ti NPs group, indicating that NPs could induce inflammation independently. Another study revealed that yttrium oxide (Y_2_O_3_ NPs) and ZnO NPs significantly increased the mRNA expression of IL‐8, ICAM‐1, and MCP‐1, while Fe_2_O_3_ NPs had no effect at the same concentration.^[^ ^183^ ^]^ These results suggest that chemical composition plays a vital role in inflammation induced by NPs. Acute‐phase proteins, another group of soluble inflammation mediators, can either initiate or support defensive and adaptive processes. They contribute to healing but can also lead to chronic inflammation.^[^ ^172^ ^]^ C‐reactive protein (CRP) is the most widely reported acute‐phase protein in inflammatory research. In Sprague‐Dawley rats, Si NPs increased CRP levels in both systemic and cardiac tissue.^[^ ^184^ ^]^ Furthermore, the complement system is also an important inflammatory mediator, though few studies currently focus on this topic.^[^ ^185^, ^186^ ^]^

**Figure 6:**
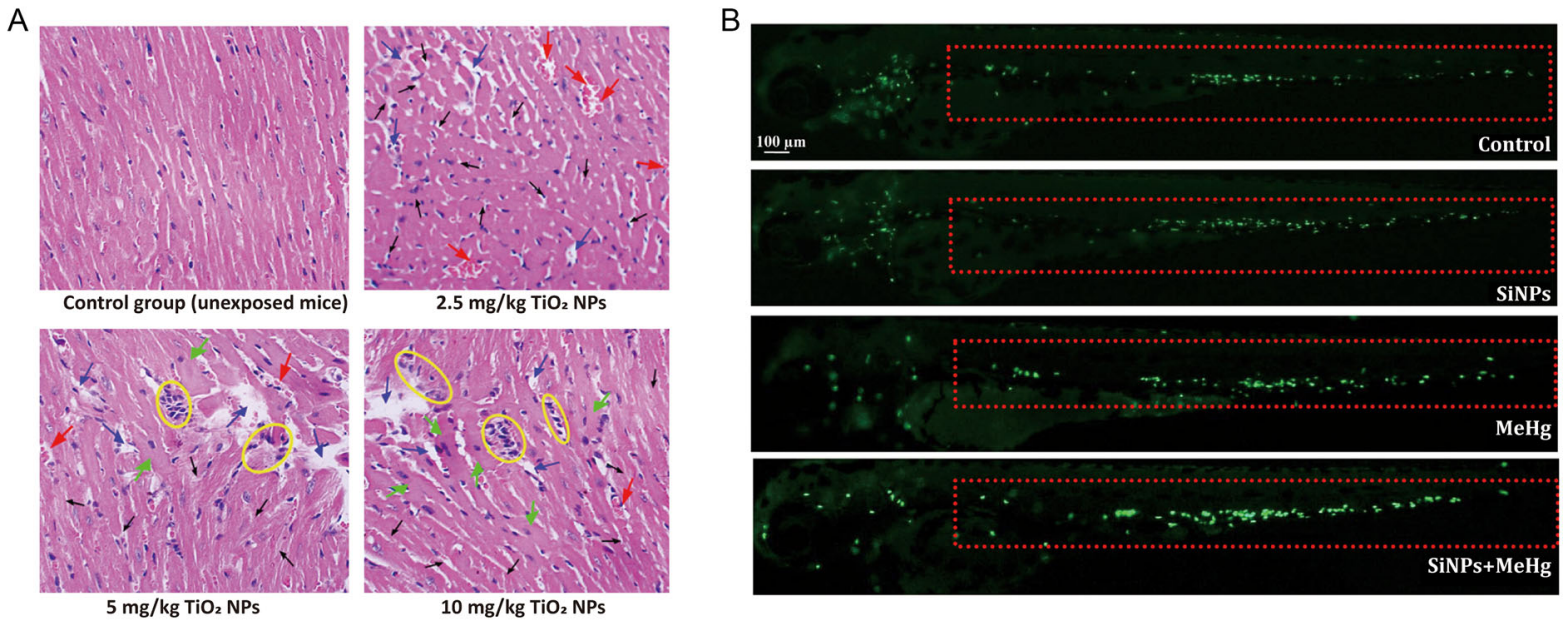
A) Histopathological changes in mouse hearts caused by intragastric administration of TiO_2_ NPs for 90 days (*n* = 5, average level of 5 mice selected randomly from each group; 400×) Reproduced with permission.^[^ ^179^ ^]^ Copyright 2016, Elsevier. B) Inflammatory response triggered by SiNPs and MeHg in Tg (mpo:GFP) zebrafish. The fluorescence of neutrophils in zebrafish caudal vein was increased gradually in the control, SiNPs, MeHg, and SiNPs + MeHg groups. Reproduced with permission.^[^ ^196^ ^]^ Copyright 2016, Elsevier.

Immune cells such as neutrophils, monocytes/macrophages, T/B lymphocytes, natural killer cells, and dendritic cells emigrate from the vasculature to damaged tissue, which is another characteristic of inflammation.^[^ ^187^ ^]^ Immune cells in cardiac tissue including those recruited from circulation and resident cells perform three roles: clearing foreign invaders and injured cells, regulating inflammatory processes, and promoting tissue repair. Macrophages phagocytoze foreign invaders and damaged cells, clear them through lysosomes, various hydrolases, ROS, and other mechanisms.^[^ ^188^, ^189^ ^]^ Inflammatory cells secrete various proteins to regulate inflammation, either by exacerbating or attenuating the adaptive immune system. The final task is to promote healing and repair. Unlike other tissues, mature cardiomyocytes have a low regenerative capacity, and resident fibroblasts become the primary cells that repair injury. After acute injury, fibroblasts accelerate matrix production to avoid catastrophic myocardial rupture.^[^ ^190^ ^]^ However, if the immune system fails to resolve the stimuli or cardiac tissue is continuously exposed to stimulation, persistent inflammation and excess matrix will lead to cardiac stiffness and electrical conduction defects, ultimately causing cardiac remodeling and heart failure.^[^ ^191^, ^192^ ^]^ For instance, cadmium (Cd) is a metal environmental biohazardous toxicant. Cd NPs significantly increased IL‐6, IL‐8, NF‐κB, and TNF‐α expression in newborn chick hearts, and histopathology revealed myocardial fiber dissolution and inflammatory cell infiltration.^[^ ^193^ ^]^ Subsequent research found that selenium (Se) NPs relieve inflammation induced by Cd NPs.^[^ ^194^ ^]^ After exposure to TiO_2_ NPs for 90 days, histopathological evaluation also showed significant cardiomyocyte injury and inflammatory cell infiltration in mice^[^ ^179^ ^]^ (**Figure** **6**A). Given the complex composition of air pollution particles, coexposure is an important topic. Zebrafish is a powerful model to explore early cardiac development and function. After coexposure to Si NPs and benzo[a]pyrene (B[a]P), a significant increase in neutrophils in the caudal vein was induced, and proinflammatory gene expression was upregulated.^[^ ^195^ ^]^ The author also explored the cardiotoxicity of coexposure to Si NPs and methylmercury (MeHg) in zebrafish, which decreased cardiac output, induced vascular endothelial damage, and increased neutrophils in zebrafish embryos^[^ ^196^ ^]^ (Figure 6B). Black carbon containing carbon NPs (CNPs) are an urban air pollutant. CNPs cross the endothelium, distribute in heart tissue, and induce apoptosis and inflammatory cell infiltration in the zebrafish heart.^[^ ^197^ ^]^

Taken together, evidence from key steps indicates that nanomaterials induce inflammation and injury in the heart. However, unlike the “first contact” organs, such as the lungs, stomach, and skin, where the relationship between NPs and the inflammatory response has been intensely investigated, the details of inflammation induced by NPs in the heart remain unclear. In the past, there were few opportunities for foreign invaders to reach the heart, so, many studies on the immune system in the heart focused on myocardial ischemia injury and repairing.^[^ ^187^ ^]^ As discussed before, many NPs can cross the endothelium into cardiac tissue. With the advancement of nanotechnology, humans will increasingly be exposed to various NPs. As the primary response to harmful stimuli, more research is needed in the future to provide a more complex perspective. Current research only explores the major steps of inflammation, while more specific and systematic evaluation of the inflammatory response to NPs in the heart is needed. Compared to oxidative stress, the effect of different NP characteristics, such as size, shape, and surface coating, on the inflammatory response in the heart is rarely reported. Future research should explore the entire inflammation process, the function of each immune cell, differences between resident and recruited leukocytes, and differences in inflammation between the heart and other organs when exposed to NPs.

## 5 Nanomaterial‐Induced Cell Death

NPs induce cell death in cardiovascular system through various pathways, including newly identified mechanisms such as necroptosis. Cell death is a complex process with multiple forms, several of which have been identified in cardiovascular system after exposure to nanomaterials. For instance, carbon black NPs have been found to initiate cell death by disrupting mitochondrial quality control.^[^ ^198^ ^]^ The traditional classification of cell death, which distinguishes between necrosis and non‐necrosis, procedural death, and the induction of inflammation, is increasingly challenged as our understanding of these processes evolves. The Nomenclature Committee on Cell Death (NCCD) has identified 12 types of regulated cell death (RCD).^[^ ^199^ ^]^ NCCD distinguishes between accidental cell death (ACD) and RCD. However, some reports have misused programmed cell death (PCD) and RCD, and specified that PCD is a physiologic form of RCD,^[^ ^199^ ^]^ whereas cell death induced by exogenous factors (e.g., NPs) should be reported as RCD rather than PCD.

Different types cell death, such as necrosis, apoptosis, pyroptosis, and ferroptosis, represent distinct physiological and pathological processes,^[^ ^200^ ^]^ making it crucial to understand these differences for developing measures to prevent nanotoxicity‐induced cell death. Cell death in the heart typically represents an irreversible loss of cardiomyocytes, which are replaced by fibroblast‐derived scar tissue, altering extracellular matrix and resulting in myocardial remodeling. The characteristics and associations between different types of cell death have been reviewed in several works.^[^ ^201^, ^202^, ^203^, ^204^, ^205^ ^]^ However, we should note that disruption of any essential process of survival may result in cell death. At the cellular level, cell death is the final outcome of multiple types of severe injury which we discussed before. However, these mechanisms do not account for all instances of NP‐induced cell death. At the tissue level, cell death represents the early stage of tissue injury, with different cell deaths contributing to tissue injury. This section outlines the primary cell death pathways triggered by NPs in CVD.

### 5.1 Necrosis

Necrosis is traditionally viewed as an uncontrolled and ACD occurring when the plasma membrane is rapidly destroyed by acute and severe stimuli, such as ischemia. However, research has found that necrosis can be regulated, known as “necroptosis,” a process involving necrosomes, including receptor‐interacting protein kinase 1 (PIPK1) and RIPK3.^[^ ^206^, ^207^ ^]^ For instance, inhaled carbon black NPs interact with lung macrophages, leading to lysosomal injury and plasma membrane rupture, causing necrosis within 30 min.^[^ ^208^ ^]^ Since the heart is not the foremost organ that comes into contact with NPs, achieving the high concentration needed to induce necrosis is difficult. Therefore, relatively few studies have investigated necrosis induced by NPs in the heart. Research has reported acute and overwhelming cell death with iron oxide/polymer nanoplatform accumulation in lysosomes, while no oxidative stress or apoptosis has been detected. With phase contrast illumination evidence, research has shown that necrosis is the only cell death type in their work.^[^ ^209^ ^]^ In another study, researchers used propidium iodide and annexin V staining to reveal that 20‐nm silicon (Si) NPs induced necroptosis and upregulated the expression of the necrosome component RIPK3.^[^ ^210^ ^]^

### 5.2 Apoptosis

NPs induced apoptosis in both the intrinsic and extrinsic pathways. Apoptosis is a regulated cell death pathway that avoids inducing inflammatory response. The key feature of apoptosis is the formation of apoptotic bodies that contain cytosol and organelles.^[^ ^205^ ^]^ Apoptosis can be triggered through intrinsic (such as mitochondrial) and extrinsic (death receptor‐mediated apoptosis) pathway. NPs can activate apoptosis by triggering death receptors or inducing oxidative stress, DNA damage, and ER stress. For example, the exogenous activation of death receptor transmembrane proteins, such as those in the TNF receptor family and Fas‐associated signaling (FAS) cell surface death receptor, leads to the initiation of apoptosis.^[^ ^211^, ^212^, ^213^ ^]^ In HAECs, ZnO NPs trigger FAS death receptors and induce apoptosis after 12 h.^[^ ^214^ ^]^ ZnO NPs also induce apoptosis in a dose‐dependent manner.^[^ ^215^ ^]^

Mitochondria and ER stress are two main intrinsic pathways that can cause apoptosis. NPs can induce extra ROS due to their high reactivity. Excessive ROS can trigger mitochondrial dysfunction, which leads to alteration of BCL2 family members, finally leading to mitochondrial membrane permeability and cytochrome c leakage. Cytochrome c binds to apoptotic peptidase activating factor 1 (APAF1), which controls the formation of apoptosomes. Apoptosome formation is the key feature of apoptosis and degrades cell components in a gentle way.^[^ ^216^ ^]^ Si NPs induce oxidative stress and mitochondrial damage in HUVECs, and the high levels of apoptosomes and cytochrome c also prove the occurrence of apoptosis.^[^ ^79^ ^]^ ZnO NPs can also induce cell apoptosis in the mitochondrial pathway with oxidative stress.^[^ ^214^ ^]^ After entering the cytosol, NPs can trigger ER stress by disrupting the process of protein folding due to their high surface reactivity.^[^ ^217^, ^218^, ^219^, ^220^, ^221^ ^]^ Additionally, oxidative stress induced by NPs may also contribute to this process.^[^ ^222^ ^]^ As a mechanism to restore cellular homeostasis, ER stress activates apoptosis if the stimuli cannot be resolved.^[^ ^216^ ^]^ After exposure to Si NPs, XBP1 splicing and GRP78/BiP (ER stress marker) and the crucial proteins (CHOP and Caspase 12) of the ER stress‐mediated apoptotic pathway were significantly increased in endothelial cells.^[^ ^79^ ^]^ This phenomenon was also reported in a human cardiac microvascular endothelial cell line after exposure to mesoporous tantalum oxide nanomaterials (PEG@mTa_2_O_5_).^[^ ^223^ ^]^

Interestingly, silica NPs disrupted gap junction intercellular communication in H9C2 cells and induced apoptosis. Enhancing the junctional channel decreased the rate of SiNP‐induced apoptosis, while inhibiting the junction channel increased SiNP‐induced apoptosis.^[^ ^224^ ^]^ This indicates that nanomaterials can induce apoptosis by disrupting the intercellular communication. In HUVECs, CuO NPs enter cells and are located in lysosomes, which degrade them. CuO NPs inhibit autophagosome‐lysosome fusion and block autophagy, finally causing apoptosis. On the other hand, the deposition of lysosomes promoted the release of Cu ions from CuO NPs, which can also induce apoptosis through oxidative stress and DNA damage.^[^ ^225^ ^]^ Similarly, the apoptosis induced by NPs was also affected by size and dose. In AC16 cells, Si NPs‐60 nm induced apoptosis in a dose‐dependent manner, and Si NPs‐6 increased apoptosis by more than twofold compared to Si NPs‐300 nm at the same dose.^[^ ^226^ ^]^

### 5.3 Pyroptosis

Pyroptosis is an inflammatory cell death that is executed by gasdermin (GSDM) family proteins. The N‐terminus of GSDMD forms a 1–2 μm pore on the cell membrane, which leads to cellular lysis and inflammatory cytokine release.^[^ ^227^, ^228^ ^]^ Current research has identified four distinct pathways that can initiate pyroptosis.^[^ ^229^ ^]^ However, current research has not explored these pathways in pyroptosis induced by NPs in the heart. However, it is clear that ion release from NPs and oxidative stress has contributed to this process. Metal oxide‐based NPs may directly activate caspase proteins, which cleave GSDMD proteins and ultimately lead to pyroptosis.^[^ ^230^ ^]^ Metal oxide‐based NPs can also release metal ions after degeneration by lysosomes and trigger pyroptosis.^[^ ^231^ ^]^ Oxidative stress also contributes to the process of pyroptosis induced by NPs. In VSMCs, hydroxyapatite NPs release Ca^2+^ after degeneration in lysosomes, and excessive Ca^2+^ disrupts mitochondrial function and promotes mitochondrial ROS production. Overproduction of ROS hurts mitochondria and promotes the release of oxidized mitochondrial DNA (Ox‐mtDNA) into the cytoplasm, which is the most effective inducing factor of pyroptosis and finally causes severe pyroptosis.^[^ ^232^ ^]^ Exposure to silicon NPs (Si NPs) via intratracheal instillation promoted inflammation, oxidative stress, and pyroptosis in rat hearts. In vitro studies have demonstrated that inhibiting NADPH oxidase can attenuate pyroptosis, while inhibiting caspase‐1 can mitigate Si NP‐induced cardiac hypertrophy.^[^ ^233^ ^]^ Another interesting working report showed that microplastics (510.4 nm) also induced pyroptosis in rat hearts.^[^ ^234^ ^]^ These results indicate that NPs can injure the heart through pyroptosis.

### 5.4 Ferroptosis

NPs, particularly iron‐based ones, can induce ferroptosis in heart tissue. Ferroptosis is an iron‐dependent form of cell death characterized by intracellular iron overload, a decrease in glutathione (GSH), and lipid peroxidation. Since phospholipids are the fundamental structure of the cell membrane, lipid peroxidation in ferroptosis causes cell membrane injury and then cell death.^[^ ^235^ ^]^ Furthermore, iron is a crucial essential ion in the heart that participates in various biological processes, while there is a complex system to maintain iron.^[^ ^236^ ^]^ Many CVD are related to iron overload or deficiency. Iron overload‐related ferroptosis was also explored in the heart, especially for iron‐based NPs. The valence of nanoiron oxides also affects the toxicity of NPs. Naying et al. synthesized three different‐valence nanoiron oxides, nFe@Fe_3_O_4_, nFe_3_O_4_, and nFe_2_O_3_, and the results showed that all NPs induced ferroptosis and defects in zebrafish embryos. These NPs also caused pericardial edema and cardiac bleeding in zebrafish. High‐valence NPs (nFe_3_O_4_ and nFe_2_O_3_) increased atrial size, while low‐valence NPs increased ventricle size in zebrafish hearts. The order of ferroptosis‐induced potential was nFe@Fe_3_O_4_ < nFe_3_O_4_ < nFe_2_O_3_, but the developmental toxicity of cardia was completely opposite.^[^ ^237^ ^]^ Interestingly, researchers found that the cardiac defects caused by nFe_3_O_4_ and nFe_2_O_3_ in zebrafish embryos can be completely rescued by ferroptosis inhibitors (Fer‐1) and iron chelators (DFO) but only partially rescued by nFe@Fe_3_O_4_. However, additional oxygen supplementation (CaO_2_) with Fer‐1 and DFO completely rescued cardiac defects induced by nFe@Fe_3_O_4_.^[^ ^237^ ^]^ Oxygen is consumed when iron converts from low‐valence iron to high‐valence states. This indicates that low‐valence iron‐based NPs (nFe@Fe_3_O_4_) caused cardiac development defects both by inducing ferroptosis and by consuming dissolved oxygen. Another widely concerning topic is PM2.5, which may release iron after inhalation into the body. In a previously mentioned postmortem study, researchers carefully explored the iron burden in heart tissue among people living in different urban areas with different levels of PM2.5. Higher levels of iron NPs (≈15–40 nm) were found in the heart tissue of people who lived in highly polluted urban areas, even in donors as young as 3 years old, compared to donors who lived in less populated and polluted locations.^[^ ^77^ ^]^ In another study, PM2.5 (SRM1648a) increased iron content and induced lipid peroxidation in both EA.hy926 cells and HUVECs.^[^ ^238^ ^]^ Magnetite iron oxides (Fe_3_O_4_) and maghemite iron oxides (γ‐Fe_2_O_3_) also increased intracellular iron ion levels and induced ferroptosis in HUVECs. Compared to γ‐Fe_2_O_3_, Fe_3_O_4_ releases more iron ions and shows greater cytotoxicity. This may indicate that iron release has a greater effect on the toxicity of NPs.^[^ ^239^ ^]^ At the same time, these NPs also disturb iron metabolism, which can lead to cellular iron accumulation.^[^ ^239^ ^]^

However, the actual conditions are quite complex. A single type of NP can activate multiple cell death pathways, and different NPs can trigger the same cell death pathway. There are observable trends in how different cell death mechanisms occur. With the low degree toxicity of NPs, the stimuli can be resolved by altering cell homeostasis, such as changing gene or protein expression, to initiate repair mechanisms, such as upregulating the antioxidant system and autophagy systems. As the toxicity of NPs increases, cells may initiate apoptosis, a process in which cells choose to undergo RCD to clear foreign stimuli. Apoptosis did not induce inflammation, which may indicate that the stimuli can be resolved at the single‐cell level. However, if the toxicity continues to increase, cell death, such as pyroptosis and ferroptosis, will occur, which can induce inflammation and neighboring cell damage. Acute and severe toxicity will cause ACD through physical, chemical, or mechanical mechanisms.^[^ ^199^ ^]^ However, there is no absolute boundary, and different types of cell death may overlap with certain stimuli. Wang et al. reported that in H9C2 cells, cell viability sharply decreased when ZnO NPs (50 nm) increased to 10 μg mL^−1^ from 2.5 μg mL^−1^ with a gradient, and apoptosis was the main cell death pathway. When the dose of ZnO NPs increased to 15 μg mL^−1^, ferroptosis became the main cell death pathway.^[^ ^215^ ^]^ In another study, necrosis increased rapidly as the ZnO NP (≈300 nm) dose increased, but apoptosis was still the main pathway of cell death even at 20 μg mL^−1^. This difference may be due to the different sizes of ZnO NPs used in the two studies.^[^ ^240^ ^]^

In summary, NPs can induce various forms of cell death. Since cardiomyocytes are incapable of regeneration after death, it is crucial to prevent NP‐induced cell death. As discussed earlier, different forms of cell death involve different signaling pathways, which places higher demands on nanomedicine design to minimize nanotoxicity.

## 6 Strategies to Mitigate Cardiovascular Toxicity of Nanomaterials

The ultimate goal of nanotoxicology is to ensure the safe use of nanomaterials, with safety being just as important as efficiency. Since most nanomedicine enter the bloodstream, cardiotoxicity is an unavoidable concern. To mitigate cardiovascular toxicity, comprehensive measures are required at every stage, from design through to application. The Organization for Economic Co‐operation and Development (OECD) has proposed “Safe(r)‐by‐Design” and “Regulatory Preparedness” as key strategies for nanomaterials to reduce uncertainties and risks to human and environmental safety.^[^ ^241^ ^]^ For Safe(r)‐by‐Design,” strategies such as selecting appropriate materials, using dopants, and employing surface coatings and functionalization have been shown to reduce nanotoxicity at the design stage. For “Regulatory Preparedness,” leading countries and regions in this field, such as USA,^[^ ^242^ ^]^ Europe,^[^ ^243^ ^]^ China,^[^ ^244^ ^]^ Japan,^[^ ^245^ ^]^ along with other international organizations,^[^ ^241^, ^246^, ^247^ ^]^ have sought to establish specialized regulation for nanoproducts, covering aspects like production, risk assessment, occupational exposure, and environmental protection. Many of these regulations highlight concerns regarding the cardiotoxicity of nanomedicine. As of August 2024, more than 900 policy documents about nanomaterials around the world have been collected in Stat Nano database.^[^ ^248^ ^]^ In general, nanomaterials are subject to more comprehensive and rigorous risk assessments. International Organization for Standardization regulations recommend that detailed characteristics of nanomaterials, such as particle size and size distribution, aggregation and agglomeration state, surface area, surface chemistry, surface charge, and redox potential, are needed for accurate risk assessment.^[^ ^249^, ^250^ ^]^ On an individual level, it is crucial to avoid accidental exposure to hazardous nanomaterials in daily life (e.g., air pollutant) and in the workplace (e.g., occupational exposure), especially for those with pre‐existing cardiovascular conditions. However, nanotoxicology is still in its early stages,^[^ ^251^ ^]^ only a few studies focus on minimizing nanotoxicity in cardiovascular system.^[^ ^252^, ^253^ ^]^ In this section, we review strategies for reducing nanotoxicity through safe design and preventing accidental exposure.

### 6.1 Modification of Physical Properties

Although lacking a mature paradigm regarding which types of materials are not harmful to cardiovascular health, certain features should also be carefully considered. A typical example is iron‐based nanomaterial with the potential to release metal ions, which should be carefully assessed. These materials not only can induce oxidative stress through the Fenton reaction, but also contribute to arrhythmia, especially for those can release Fe^2+^ and Cu^2+^.

Second, as the size decreased, the specific surface area increased, more surface defects and active sites are exposed, which result in severe toxicity.^[^ ^254^ ^]^ Due to the continuous endothelium and overlapping of cell junctions, ≈4 nm gaps between endothelial cells are observed in mice.^[^ ^45^ ^]^ However, literature reports indicated that materials larger than 1.8–2.0 nm have difficulty passing through the gap between continuous endothelia.^[^ ^255^ ^]^ Although these gaps may enlarge under pathological conditions and exposure to certain nanomaterials, the gaps in heart tissue are still significantly smaller than those found in cancer tissue.^[^ ^256^ ^]^ Therefore, if feasible, increasing the size of the products can reduce the leakage of nanomaterials into the heart through the paracellular pathway. However, these strategies to modify endocytosis are only suitable for products that do not need to enter cells, such as contrast agent. Endocytosis is also an important route for entry into target cell outside of the heart. Regrettably, the use of endocytosis inhibitors in nanomedicine to reduce unintended entry into cardiomyocytes has not been reported.

The shape of the nanomaterials also significantly alters its toxicity. This phenomenon may relate to different binding capacity of various shapes. Early research revealed that gold nanorods exhibited more severe toxicity than gold NPs. However, further exploration revealed that the toxicity is contributed by residual surfactant hexadecyl cetyltrimethylammonium bromide, which assists in nanorod growth during synthesis.^[^ ^257^ ^]^ Under physiological flow conditions, rod‐like mesoporous silica NPs exhibit better cell uptake, while spherical mesoporous silica NPs show higher cardiovascular toxicity.^[^ ^258^ ^]^ This result may be due to the fact that rod‐like shape provides a higher membrane binding ability for uptake, while the spherical shape offers a higher specific surface area for oxidative stress.^[^ ^259^, ^260^ ^]^ For carbon nanomaterials, nanotubes exhibit more severe toxicity compared to other shapes.^[^ ^261^ ^]^ Among marine microalgae, the toxicity of carbon is order as multiwalled carbon nanotubes (MCNTs) > graphene oxide (GeO) > graphene (Gr) > fullerene (c60).^[^ ^262^ ^]^ In human lymphatic endothelia cells, MCNTs suppress cell growth, while carbon black does not. However, the evidence regarding how the length of carbon nanotubes affects nanotoxicity is interesting. At the microlevel, evidence from histopathological suggests that carbon nanotubes with long length cannot be eliminated by the immune system, resulting in granuloma formation.^[^ ^263^, ^264^ ^]^ At the nanolength, shorter MCNTs induce more DNA strand breaks, while longer nanotube does not.^[^ ^265^ ^]^

### 6.2 Doping with Other Materials

Doping is a commonly used strategy in nanotechnology to improve and alter material properties.^[^ ^266^ ^]^ In rodent and zebrafish models, Xia et al. doped iron into ZnO NPs, which resulted in reduced inflammation in the lung of mice and improved embryo hatching of zebrafish.^[^ ^267^ ^]^ Further exploration revealed that iron doping reduced particles dissolution and Zn^2+^ release, which are the main contributors to ZnO NPs toxicity.^[^ ^267^, ^268^ ^]^ This result also has been observed by doping iron into CuO NPs. Although iron doping increases the cellular uptake of CuO NPs, cell death decreases as the percentage of iron doping increases. The Raman spectrum revealed that this is related to the formation of the CuFe_2_O_4_ phase on the surface CuO NPs, which prevents Cu^2+^ release from Fe‐doped CuO NPs.^[^ ^269^ ^]^ Differently, doping ZnO into MnO_2_ NPs reduced both the cellular uptake and toxicity.^[^ ^270^ ^]^ However, materials doping can also increase the toxicity of NPs. Doping Cu into ZnO NPs increased oxidative stress in macrophages, and the toxicity increased as the percentage of Cu increased.^[^ ^271^ ^]^

### 6.3 Surface Functionalization

Surface modification techniques, such as surface coating and functionalization, have been widely used to construct nanoproducts. Surface modification aims to alter or impart new surface properties for functionalization, targeting ability, biocompatibility, and other relevant attributes. Surface coating with biocompatibility materials can shield surface active sites, alter surface charge, prevent particle aggregation, and reduce cellular uptake, ultimately reducing the toxicity of NPs. Coating certain metal oxides (CoO, CuO, Ni_2_O_3_, and Co_3_O_4_) with ethylenediamine tetra (methylene phosphonic acid, EDTMP) can reduce the pulmonary inflammation caused by these NPs.^[^ ^272^ ^]^ Coating ZnO NPs with PEGylation reduced the formation of protein corona and cellular uptake.^[^ ^273^ ^]^ Chitosan‐coated ZnO NPs also demonstrated minimal cardiotoxicity even at a high concentration.^[^ ^274^ ^]^ Protein corona is also an alternative method of NP surface coating. Many researchers have incubated NPs with specific serum or proteins in vitro to promote the formation of protein corona.^[^ ^275^ ^]^ The proteins bind and occupy the active site of NP and reduce the reaction capability of NPs after administration.^[^ ^276^ ^]^ The cell membrane is another widely studied material for NP surface coating.^[^ ^277^ ^]^ Natural‐derived cell membranes avoid the concerns about biocompatibility and immunogenicity. Plasma membranes from red blood cells, stem cells, immune cells, cancer cells, extracellular vesicles, and so on were utilized to coat NPs.^[^ ^278^ ^]^ Hence, the primary objective of surface coating strategies is to minimize the interaction between NPs and cell membranes, effectively preventing cellular uptake and ultimately mitigating nanotoxicity.

### 6.4 Use of Antidotes

The strategies of using antidote include carrying antidote on nanomaterials and using exogenous antidote. The use of exogenous antidotes targeting toxicity mechanisms such as oxidative stress and inflammation is not only widely practiced in clinical settings but also extensively utilized in laboratories to identify toxicity mechanisms.^[^ ^279^, ^280^ ^]^ However, since only several nanomedicines have been used in clinically, reports of using antidote to reduce nanotoxicity in clinics are also rare. Stevanovic et al. encapsulated antioxidant vitamin C with Ag NPs to reduce ROS generation.^[^ ^281^ ^]^ Similarly, ion release is a significant contributor to cardiotoxicity, and ion chelator also has been widely employed to migrate this effect.^[^ ^149^, ^282^ ^]^ For example, the iron chelator Deferoxamine is used to chelate ion release from NPs.^[^ ^149^ ^]^ Another interesting study was carried out by Hao Fu and his colleagues, where they utilized polyacrylic acid to coat Fe_3_O_4_ nanomaterials (PION) to develop an MRI contrast agent.^[^ ^129^ ^]^ However, in vivo examinations revealed severe heart failure. Upon further investigation, it was found that PION absorbed serum Ca^2+^ and Mg^2+^, leading to ion imbalance and cardiotoxicity. Subsequently, they encapsulated Ca^2+^ into PION, successfully mitigating its cardiotoxic effects.^[^ ^129^ ^]^

### 6.5 Preventing Accidental Exposure

As the saying goes, “Anything can be a poison.” Therefore, minimizing accidental exposure to the heart is important. For medications not intended for cardiac purposes, opting for local administration over systemic administration and enhancing targeting efficiency can effectively reduce cardiac exposure. Nano‐based techniques, such as controlled releases and improved target efficiency, have been employed to reduce the accumulation of anticancer drugs in the heart. These strategies may also be applicable to nanomaterials themselves. Timing is crucial when using nanomedicine because the heart's sensitivity to NPs is not constant. For example, the gap between endothelial cells can widen under certain pathological conditions and result in increasing accumulation in heart.^[^ ^283^ ^]^ Nanotoxicity is not solely determined by NP properties; the cellular state also plays a crucial role. The ratios of proteins, lipids, and glucose levels also can influence the interaction between NPs and cell membranes. For instance, researchers have revealed that unmodified silica NPs can disrupt the membrane when they exhibit a low lipid headgroup tilt angle and contain a trimethylammonium moiety, while carboxyl‐modified silica NPs only disrupt lipids with cis unsaturation and a sphingosine backbone.^[^ ^284^ ^]^ This highlights that more caution is needed when using nanomedicines, such as contrast agents, in patients with specific CVD.

Avoiding accidental environmental exposure to nanomaterials is a useful strategy for individuals to mitigate nanotoxicity. Prestudies in air pollution provide strong evidence that reducing NPs pollutant exposure through the use of personal protective equipment can significantly decrease the risk of CVD. For industries, adopting environmentally friendly synthesis methods, providing protective equipment for occupational workers, and reducing emissions of nanosized pollutants are crucial measures.

In summary, although methods are limited, several strategies can still be employed to reduce cardiotoxicity associated with nanomaterials. Selecting biocompatible materials, optimizing size and shape, limiting ion release, enhancing targeting capabilities, and inhibiting surface activity of the final nanoproducts are effective measures to mitigate nanotoxicity. For nanoproducts that do not target the cardiovascular system, adjusting the administration route to minimize cardiovascular exposure is practical approaches to avoid cardiotoxicity. For nanoproducts specifically targeting the cardiovascular system, strategies such as reducing ion release, lowering oxidation capacity, and promoting excretion can help minimize cardiotoxicity. However, these strategies may sometimes conflict with the primary design goals of the nanoproducts, necessitating specific analysis customization for each product. Unlike other organs, the impact on arrhythmias is a critical factor that sets cardiovascular toxicity apart from general toxicity mechanisms, requiring additional attention. While existing guidelines on the risk of arrhythmias for traditional products^[^ ^285^, ^286^ ^]^ may offer valuable insights, specific guidelines for nanomaterials are currently lacking. Additionally, there remains a significant gap in our understanding of the cardiotoxicity induced by nanomaterials. Although current regulations address nanocardiotoxicity, a systematic risk assessment protocol for cardiotoxicity is still lacking. Further research is essential to validate these strategies and to develop more robust risk assessment protocols for mitigating cardiotoxicity.

## 7 Conclusion and Future Perspectives

Cardiotoxicity induced by nanomaterials has garnered great attention over the past decades. Bibliometric analysis allows for a quantitative assessment of the research landscape and developments of nanocardiotoxicity field. Based on the analysis of publications, annual growth, contributing countries/institutions, and hotspot identification, the most contributed countries/institutions including China and Chinese Academy of Sciences, and nanocardiotoxicity mechanisms including biodistribution, oxidative stress, inflammation, and cell death are recognized. Drawing insights from the bibliometric analysis, we then reviewed the pathways through which nanomaterials enter the heart and the mechanisms underlying nanotoxicity in cardiac tissue. Nanomaterials reach the heart via various administration and exposure routes, entering cells through passive diffusion and endocytosis and localizing in different organelles. During these processes, nanomaterials adhere to cell membranes, leading to cell injury and disruption of ion channels. Once inside the cell, they can induce oxidative stress, inflammation and trigger cell death—fundamental mechanisms driving cardiotoxicity. The goal of reducing cardiotoxicity should be considered from the design stage. Strategies such as surface modification, doping, the use of antidotes to enhance biocompatibility, limiting ion release, and preventing accidental cardiac exposure are also summarized and proposed to mitigate nanotoxicity.

Nanomaterials designers face significant challenges. Despite the rapid advancement of nanotechnology, our understanding of nanotoxicity, particularly in cardiovascular system, remains limited. Strategies to reduce cardiotoxicity are also scarce. Current evidence is sometimes conflicting, with certain studies claim the safety of specific nanomaterials, while others do not. Therefore, detailed characterization of nanomaterials is necessary in future studies, encompassing not only their in vitro properties but also their in vivo biological behaviors, which must be carefully tracked. Additionally, while guidelines for assessing nanotoxicity in other areas have been published,^[^ ^287^ ^]^ there remains a need for detailed guidelines specifically addressing nanotoxicity in the cardiovascular system. Another challenge is the insufficiency of biological models. Lipid bilayers, commonly used to study interactions between cell membranes and NPs, do not fully replicate the complexity and dynamics of cell membranes. Cardiac organoids offer promising platforms for toxicity research.

The current review focuses on the exposure routes and mechanism of nanocardiotoxicity, while previous reviews address nanomaterials classification in terms of cardiosafety including but not limited to CNP, metallic and metallic oxide NP, and nonmetallic.^[^ ^288^, ^289^, ^290^, ^291^ ^]^ However, with so many comprehensive reviews and understanding of the nanotoxicity in cardiovascular system, there are still a lack of user‐friendly databases to organize and retrieve such information. For instance, the Nanodatabase (https://nanodb.dk/en) provides a valuable example by categorizing consumer goods containing nanomaterials based on exposure risks and potential effects, making it a useful resource for the general public. A similar database that categorizes nanomaterials based on chemical composition, toxicological mechanisms, or adverse health effects would be highly beneficial. Such a resource would help researchers screen for safer nanomaterials and assist clinicians in assessing the potential risks of nanomedicines. In particular, with the rapid advancement of artificial intelligence technology, the collection and organization of data would be more efficient.

In summary, further research is needed for advancing cardiovascular‐friendly nanomedicine. The development of biological models both in vivo and in vitro will be helpful for accurately assessing cardiotoxicity. To design safety nanomedicine, collaboration among experts from nanotechnology, cardiology, nanotoxicology, clinical practice, and regulatory bodies is needed at the early stage. Comprehensive and practical regulations regarding cardiotoxicity assessment and postmarketing surveillance are equally necessary. With the development of advanced models and novel methods, we believe nanocardiotoxicity can be significantly reduced, paving the way for the safe and effective use of nanomaterials in cardiovascular medicine.

## Conflict of Interest

The authors declare no conflict of interest.

## Author Contributions

**Zhanjun Gu**: conceptualization: (lead); funding acquisition: (equal); supervision: (lead); writing—review & editing: (lead). **Zaiyong Zheng**: data curation: (lead); formal analysis: (lead); methodology: (lead); visualization: (lead); writing—original draft: (lead). **Shuang Zhu**: data curation: (equal); formal analysis: (equal); project administration: (supporting); writing—review & editing: (lead). **Xiaobo Wang**: formal analysis: (equal); visualization: (equal); writing—review & editing: (equal). **Haoran Wu**: formal analysis: (supporting); writing—review & editing: (supporting). **Min Fu**: visualization: (supporting); writing—review & editing: (supporting). **Houxiang Hu**: funding acquisition: (equal); supervision: (equal); writing—review & editing: (supporting). **Chunxiang Zhang**: funding acquisition: (lead); supervision: (lead); writing—review & editing: (equal). **Zaiyong Zheng**, **Shuang Zhu**, **Xiaobo Wang** contributed equally to this work.

## Supporting information

Supplementary Material
