# Supplementary material for: Understanding the Mechanism of Cardiotoxicity Induced by Nanomaterials: A Comprehensive Review
Source: Small Sci. 2025 Feb 20;5(5):2400498. doi: 10.1002/smsc.202400498 (PMC12087784; doi:10.1002/smsc.202400498)
Supplement: Supplementary file 1 — Supplementary Material [file SMSC-5-2400498-s001.pdf]

Supporting Information

Understanding the Mechanism of Cardiotoxicity Induced by Nanomaterials: A Comprehensive Review

Zaiyong Zheng, Shuang Zhu, Xiaobo Wang, Haoran Wu, Min Fu, Houxiang Hu, Zhanjun Gu*, Chunxiang Zhang**

Z.Y. Zheng, S. Zhu, and X.B. Wang contributed equally to this work.
Z.Y. Zheng, H.R. Wu, M. Fu, C.X. Zhang
Department of Cardiology, Key Laboratory of Medical Electrophysiology, Ministry of Education; Basic Medicine Research Innovation Center for cardiometabolic diseases, Ministry of Education; The Affiliated Hospital of Southwest Medical University, Southwest Medical University,
Luzhou, 646000, China
Email: zhangchunxiang@swmu.edu.cn
S. Zhu, Z.J. Gu
CAS Key Laboratory for Biomedical Effects of Nanomaterials and Nanosafety, Institute of High Energy Physics, Beijing 100049, China
S. Zhu
Spallation Neutron Source Science Center, Institute of High Energy Physics, Dongguan 523803, China
Z.J. Gu
College of Materials Science and Optoelectronic Technology, University of Chinese Academy of Sciences,
Email: zjgu@ihep.ac.cn
Z.Y. Zheng, X.B. Wang, H.X. Hu
Academician Workstation and Department of Cardiology, Affiliated Hospital of North Sichuan Medical College,
Nanchong 637000, China
Email: hhxiang@nsmc.edu.cn
Keywords: Nanomaterials; Nanotoxicity; Cardiovascular health; Bibliometric analysis

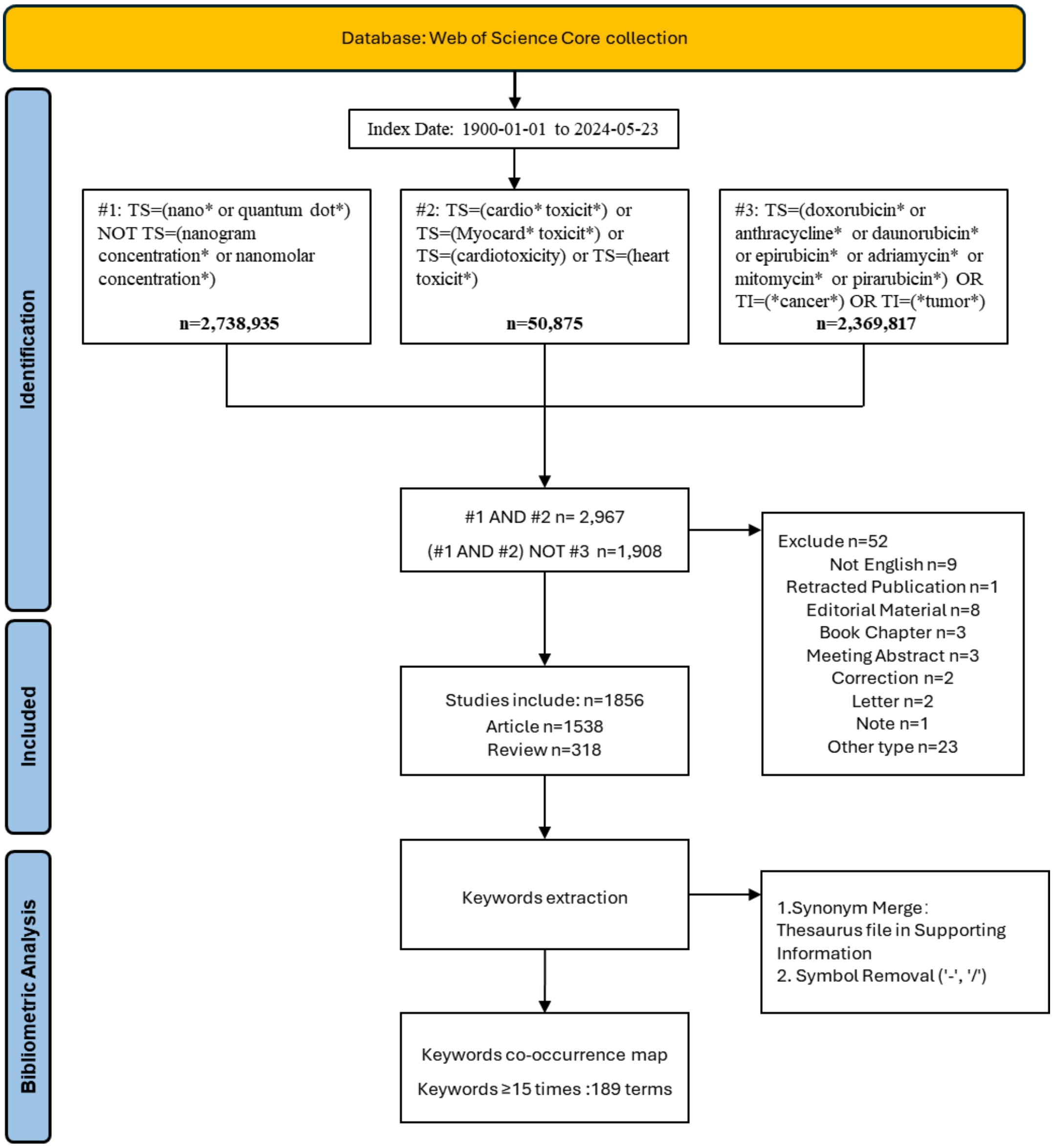

Figure S1. The flow diagram indicating the recruiting criteria of current study.

Table S1. Dictionary of Synonym Replacements.

Label	Replace by
a549	a549 cells
aluminum oxide nanoparticles	aluminum oxide
amorphous silica nanoparticles	amorphous silica
antioxidant enzymes	antioxidant enzyme
aqueous solutions	aqueous solution
arrhythmias	arrhythmia
assays	assay
associations	association
benefits	benefit
biomarkers	biomarker
biomaterials	biomaterial
biomedical applications	biomedical application
biosensors	biosensor
carbon based nanomaterials	carbon based nanoparticles
carbon nanoparticles	carbon nanoparticle
carbon nanotubes (cnts)	carbon nanotube
carbon nanotubes (cnts)	carbon nanotube
cardiac patch	cardiac patches
cardiovascular risk factors	cardiovascular risk
cell line	cell lines
chicken	chickens
chitosan nanoparticles	chitosan nanoparticle
contrast agents	contrast agent
cyclodextrins	cyclodextrin
dendrimers	dendrimer
diabetes	diabetes mellitus
diagnostics	diagnosis
drug carriers	drug carrier
emulsions	emulsion
engineered nanomaterials	engineered nanoparticles
enzymes	enzyme
exosomes	exosome
extracellular vesicles	extracellular vesicle
films	film
fine particles	fine particle
fine particulate matter	fine particle
flavonoids	flavonoid
formulations	formulation
growth factors	growth factor
health risks	health risk
heart diseases	heart disease
human endothelial cells	human endothelial cell
human umbilical vein endothelial cells (huvecs)	human umbilical vein endothelial cell
huvec	human umbilical vein endothelial cell
huvecs	human umbilical vein endothelial cell
hydrogels	hydrogel
pluripotent stem cells	pluripotent stem cell

Table S1. Dictionary of Synonym Replacements (Continued).

Label	Replace by
inflammatory responses	inflammatory response
inhibitors	inhibitor
iron oxide nanoparticles	iron oxide nanoparticle
lipid emulsions	lipid emulsion
lipoproteins	lipoprotein
liposomes	liposome
liver x receptors	liver x receptor
mast cells	mast cell
matrix metalloproteinase 2	matrix metalloproteinases
mechanisms	mechanism
mesoporous silica nanoparticles (msns)	mesoporous silica nanoparticles
meta analysis	metaanalysis
metabonomics	metabolomics
metal organic frameworks	metal organic framework
metallothioneins	metallothionein
micelles	micelle
microplastics	microplastic
micrornas	microrna
molecular mechanisms	molecular mechanism
multi walled carbon nanotubes (mwcnts)	multi walled carbon nanotube
multiwalled carbon nanotubes	multiwall carbon nanotube
mwcnt	multiwall carbon nanotube
mwcnts	multiwall carbon nanotube
nanocomposites	nanocomposite
nanocrystals	nanocrystal
nanoformulations	nanoformulation
nanomaterials	nanomaterial
nanoprobes	nanoprobe
nanosuspensions	nanosuspension
nanotubes	nanotube
networks	network
neurodegenerative diseases	neurodegenerative disease
neurotransmitters	neurotransmitter
pamam dendrimers	pamam dendrimer
pathways	pathway
pharmacokinetics	pharmacokinetic
phytochemicals	phytochemical
plaques	plaque
pluripotent stem cells	pluripotent stem cell
polymeric nanoparticles	polymeric nanocapsules
polyphenols	polyphenol
polystyrene nanoplastics	polystyrene nanoparticles
probes	probe
prodrugs	prodrug
pulmonary responses	pulmonary response
quantum dot nanoparticles	quantum dots
quantum dot	quantum dots

Table S1. Dictionary of Synonym Replacements (Continued).

Label	Replace by
reactive oxygen species (ros)	reactive oxygen specie
receptors	receptor
respiratory diseases	respiratory disease
reactive oxygen species	ros
reactive oxygen species	ros production
scaffolds	scaffold
sensors	sensor
signaling pathways	signaling pathway
sio2	sio2 nanoparticles
solid lipid nanoparticles (sln)	solid lipid nanoparticles
superparamagnetic particles	superparamagnetic nanoparticles
surfactants	surfactant
swcnt	single walled carbon nanotube
swcnts	single walled carbon nanotube
theranostics	theranostic
thermosensitive hydrogels	thermosensitive hydrogel
transcription factors	transcription factor
wall carbon nanotubes	walled carbon nanotubes
zebrafish embryos	zebrafish embryo
zinc oxide nanoparticles	zinc oxide nanoparticle
3d printing	3d bioprinting
acute lung injury	acute lung toxicity
acute myocardial ischemia	myocardial infarction
acute toxicity evaluation	acute toxicity
adult zebrafish	zebrafish
agents	agent
agglomeration	aggregation
air pollution exposure	air pollution
alzheimer	alzheimers disease
alzheimer's disease	alzheimers disease
amyloid beta	amyloid beta peptide
amyloid beta	amyloid beta peptide
animal models	animal model
antibacterial activity	antibacterial
anticancer agents	anticancer drug
anticancer drugs	anticancer drug
antimicrobial peptides	antimicrobial peptide
antioxidant activity	antioxidant
antioxidant capacity	antioxidant
antioxidant properties	antioxidant
antioxidation	antioxidant
antitumor activity	antitumor efficacy
antitumor	anticancer
aquatic toxicity	aquatic toxicology
bio distribution	biodistribution
biological activities	biological activity
block copolymers	block copolymer

Table S1. Dictionary of Synonym Replacements (Continued).

Label	Replace by
cardio toxicity	cardiac toxicity
cardiomyoblasts	cardiomyoblast
cardiomyocytes	cardiomyocyte
cardioprotection	cardioprotective
cardiomyogenic differentiation	cardiomyocyte differentiation
cardiovascular diseases	cardiovascular disease
cardiovascular disorders	cardiovascular disease
cardiovascular effects	cardiovascular effect
cardiovascular toxicity	cardiac toxicity
carriers	carrier
chemical constituents	chemical composition
composites	composite
conjugate	conjugation
controlled drug release	controlled release
copolymers	copolymer
coronary artery disease	coronary heart disease
cuo nanoparticles	cuo nanoparticle
cytochrome c release	cytochrome c
delivery systems	delivery system
delivery vehicles	delivery system
developmental toxicity	development toxicity
doxorubicin (dox)	doxorubicin
drug delivery system	drug delivery
drug delivery systems	drug delivery
drugs	drug
embryotoxicity test	embryotoxicity
endothelial cell (ec)	endothelial cell
endothelial cells	endothelial cell
environmental toxicity	environmental toxicology
extraction	extract
extracts	extract
fluorescent	fluorescence
fullerenols	fullerenol
fullerol	fullerenol
gene expression changes	gene expression
glycol)	glycol
gold nanoparticles	gold nanoparticle
gold nanoparticles (aunps)	gold nanoparticle
heme oxygenase 1	heme oxygenase
hyaluronic acid (ha)	hyaluronic acid
immunotoxicity	immunotoxicology
impacts	impact
in vitro characterization	in vitro
in vitro cytotoxicity	in vitro
in vitro evaluation	in vitro
in vitro model	in vitro
in vitro toxicity	in vitro

Table S1. Dictionary of Synonym Replacements (Continued).

Label	Replace by
in vitro toxicology	in vitro
in vivo biodistribution	biodistribution
in vivo delivery	in vivo
in vivo evaluation	in vivo
in vivo fate	in vivo
in vivo genotoxicity	in vivo
in vivo toxicity	in vivo
inflammatory	inflammation
inhalation toxicity	inhalation toxicology
ischemia reperfusion injury	ischemia reperfusion
ischemia/reperfusion	ischemia reperfusion
ischemia/reperfusion injury	ischemia reperfusion
macrophage membrane	membrane
magnetic resonance imaging (mri)	magnetic resonance imaging
membranes	membrane
messenger rna expression	messenger rna
microarrays	microarray
mitochondria	mitochondrion
mitochondrial dysfunction	mitochondrial damage
mitochondrial dna damage	mitochondrial damage
mitochondrial	mitochondrion
mitoxantrone (mtx)	mitoxantrone
mri	magnetic resonance imaging
myocardial toxicity	cardiac toxicity
nano medicine	nanomedicine
nanobiomedicine	nanomedicine
nanoliposome	liposome
nanomedicines	nanomedicine
nanodrugs	nanomedicine
nanoparticles	nanoparticle
nanosilver	nano silver
nanotechnologies	nanotechnology
oxidatively damaged dna	oxidative dna damage
parkinson's disease	parkinsons disease
physicochemical characteristics	physicochemical characterization
corona	protein corona
proteins	protein
resistance	resistant
rna seq	rna sequencing
silica nanoparticle (sinp)	silica nanoparticle
silica nanoparticles	silica nanoparticle
silver nanoparticles	silver nanoparticle
silver nanoparticles (agnps)	silver nanoparticle
stem cells	stem cell
systems	system
targeted delivery	targeted drug delivery
targets	target

Table S1. Dictionary of Synonym Replacements (Continued).

Label	Replace by
transfection efficiency	transfection
tumor necrosis factor alpha	tumor necrosis factor
vascular endothelial cell death	vascular endothelial cell
vascular endothelial cells	vascular endothelial cell
zebrafish (danio rerio)	zebrafish
zebrafish danio rerio	zebrafish
nanotoxicity	nanotoxicology
antioxidants	antioxidant
carbon nanotubes	carbon nanotube
cardiotoxicity	cardiac toxicity
ecotoxicity	ecotoxicology
macrophages	macrophage
nanoparticles	nanoparticle
acute myocardial infarction	myocardial infarction
agnps	silver nanoparticle
biodegradable	biodegradation
c 60(oh)(24)	c 60
embryos	embryo
fullerenes	fullerene
genes	gene
drug-delivery	drug delivery
myocardial-infarction	myocardial infarction
DNA-DAMAGE	dna damage
MOLECULAR-MECHANISM	molecular mechanism
CARDIOVASCULAR-RESPONSES	cardiovascular responses
in-vivo	in vivo
vivo	in vivo
in-vitro	in vitro
vitro	in vitro
air-pollution	air pollution
cells	cell
endothelial-cells	endothelial cell
gene-expression	gene expression
heart-failure	heart failure
nitric-oxide	nitric oxide
rats	rat
stem-cells	stem cell
